# Luminescence Thermometry Based on Time Gates: Highly Sensitive Approach for Real-Time Sensing and Imaging

**DOI:** 10.1021/acs.jpclett.5c01265

**Published:** 2025-06-06

**Authors:** M. Szymczak, D. Szymański, M. Piasecki, M. Brik, L. Marciniak

**Affiliations:** † 215275 Institute of Low Temperature and Structure Research, Polish Academy of Sciences, Okólna 2, 50-422 Wrocław, Poland; ‡ Faculty of Science and Technology, Jan Długosz University, Armii Krajowej 13/15, 42-200 Częstochowa, Poland; § School of Optoelectronic Engineering, Chongqing University of Posts and Telecommunications, Chongqing 400065, China; ∥ Centre of Excellence for Photoconversion, Vinča Institute of Nuclear Sciences - National Institute of the Republic of Serbia, University of Belgrade, 11351 Belgrade, Serbia; ⊥ Institute of Physics, 37546 University of Tartu, W. Ostwald Str. 1, 50411 Tartu, Estonia; # Academy of Romanian Scientists, Ilfov Str. No. 3, 050044 Bucharest, Romania

## Abstract

Undoubtedly, one of the most significant advantages of luminescence thermometry is its ability to be used not only for spot temperature measurements but also for imaging temperature changes. Among the commonly proposed approaches, luminescence thermometry based on luminescence kinetics holds particular promise. However, most thermometric studies rely on the analysis of luminescence decay profiles, a method that significantly hinders, if not entirely precludes, real-time thermal imaging. In this Letter, we propose an alternative approach based on the luminescence intensity ratio integrated over two temporal gates. Tests conducted on two representative phosphors, Ba_2_LaNbO_6_:1%Mn^4+^ and Ca_2_LaNbO_6_:1%Mn^4+^, demonstrate that the proposed method not only enables thermal imaging but also achieves substantially higher relative sensitivity, reaching *S*_R_ = 17.1% K^–1^ for Ba_2_LaNbO_6_:1%Mn^4+^ and *S*_R_ = 9.4% K^–1^ for Ca_2_LaNbO_6_:1%Mn^4+^, compared to the conventional lifetime-based approach (*S*_R_ = 4.2% K^–1^ for Ba_2_LaNbO_6_:1%Mn^4+^ and *S*_R_ = 1.2% K^–1^ for Ca_2_LaNbO_6_:1%Mn^4+^). Furthermore, the careful selection of gate lengths allows optimization of the thermometric performance of the proposed luminescent thermometers. This approach enables expansion of the thermal operating range at the cost of relative sensitivity, providing versatility to adapt the thermometer for specific applications.

## Graphical abstract

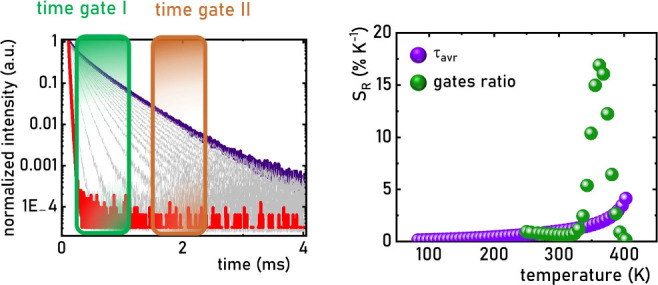

While numerous spectroscopic parameters of phosphor exhibit sensitivity to temperature changes and can therefore be utilized in luminescence thermometry, ratiometric and lifetime-based approaches are undoubtedly the most widely employed.^1–9^ This is due to several key advantages. Most notably, these methods provide highly reliable temperature readings, exhibit insensitivity to other experimental parameters (unlike intensity-based approaches), and do not require high-resolution spectrometers (as needed in spectral shift- or bandwidth-based approaches).^2,10^ Furthermore, both techniques enable not only point-based thermal readouts but also imaging of temperature distributions.^11–15^ The effectiveness of the ratiometric approach has been well-demonstrated, both using bandpass optical filter sets^14,16^ and in camera-only approaches^12^ leveraging the relationship between images captured by the RGB channels of a standard sensor. This capability is a significant advantage, as it allows the use of low-cost cameras, including smartphones, for temperature readout. However, as many studies have shown, the dispersive nature of the extinction coefficient of the medium in which the phosphor is located—or that exists in the optical path between the detector and the luminescence thermometer—can distort the phosphor’s emission spectrum, potentially compromising the reliability of the readings.^10^ Dynamic factors such as the humidity or gas content in the optical path can further complicate this issue, making in situ thermometer recalibration ineffective in many cases.

For these reasons, a lifetime-based approach may offer a better solution.^2,6,10^ In this approach, detection occurs at a single emission wavelength and the thermometer parameter is derived from the temporal evolution of the luminescence signal. As consistently demonstrated, luminescence kinetics is highly effective for temperature sensing applications,^17–19^ and recent reports have demonstrated that acquisition times as short as tens of milliseconds are sufficient to obtain a lifetime-based thermal readout.^20^ However, the sensitivity of the lifetime-based method is typically lower than that of its ratiometric counterpart^3,6^ Moreover, while lifetime-based thermometry is popular in the literature, it is predominantly reliant on luminescence decay profile measurements.^21–27^ This approach, while effective, precludes real-time thermal imaging, as it necessitates point-by-point measurements of the luminescence decay profile, which are exceedingly time-consuming.

To address these limitations, this study explores a ratiometric approach to luminescence kinetics based on a time-gated temperature determination. This study explores the capabilities of this approach without presenting a proof-of-concept experiment. Nonetheless, its efficacy in temperature measurement has been previously validated.^28^ Two phosphors doped with Mn^4+^ ions were used as representative examples: Ba_2_LaNbO_6_:1%Mn^4+^ and Ca_2_LaNbO_6_:1%Mn^4+^. Although the proposed approach is universal and can be used for any type of phosphor, there are several important advantages of materials doped with Mn^4+^ ions that facilitate the demonstration of the advantages of this solution.^29–38^ A notable advantage of Mn^4+^ ions in this context is that their ^2^E → ^4^A_2_ electron transition is spin-forbidden, resulting in a long luminescence decay profile. Additionally, the luminescence of Mn^4+^ ions is characterized by a high intensity. Moreover, the change in the strength of the crystal field interacting with Mn^4+^ ions enables modification of the rate of thermal shortening of the ^2^E state.^31–36,39,40^ In this approach thermal sensing or/and imaging of the analyzed object covered with a luminescence thermometer can be determined by the analysis of the luminescence intensity integrated into two temporal gates. The use of a spatial detector like a digital camera enables the two luminescence maps to be captured. Their luminescence intensity ratio can be easily transformed to thermal maps using the calibration curve. Comparative analysis of relative sensitivities using τ_avr_-based methods and the intensity ratio approach recorded in time gates demonstrates the superiority of the latter. Furthermore, this study highlights how the thermometric performance of such a thermometer can be modulated and optimized by adjusting the timing of the gates and the delay between them. The approach of utilizing the ratio of luminescence intensities recorded at two distinct time gates was already extensively employed in imaging various physical and chemical parameters.^41–43^ However, its application in luminescence thermometry remains relatively uncommon. Previous studies have explored this method with phosphors doped with Cr^3+^ ^44,45^ and lanthanide ions.^45,46^ The high relative sensitivity values achieved with Mn^4+^-doped phosphors, as presented in this work, render this approach particularly promising for practical applications.

## Experimental Section

### Synthesis

Phosphors Ba_2_LaNbO_6_ and Ca_2_LaNbO_6_ doped with 1% Mn^4+^ ions were synthesized using a conventional, high-temperature solid-state reaction method. The dopant ion concentration was selected arbitrarily based on our previous experience to balance high emission intensity and absorption cross section while minimizing the risk of structural defects arising from ionic charge mismatches between the dopant and host cations.

Stoichiometric amounts of Ba­(NO_3_)_2_ or CaCO_3_ (both with 99.999% purity, Thermo Scientific Chemicals), La_2_O_3_ (99.99% purity, Stanford Materials Corporation), Nb_2_O_5_ (99.9985% purity, Thermo Scientific Chemicals), and MnCl_2_·4H_2_O (>99.0% purity, Sigma-Aldrich) were carefully weighed, with the amount of the MnCl_2_·4H_2_O calculated relative to the Nb^5+^ ions in the host material. The powders were thoroughly ground in an agate mortar, using 5 mL of hexane (3 times) to ensure better homogeneity of the mixture. The resulting powders were then transferred to alumina crucibles and calcined in air at 1573 K for 6 h, with heating rate of 10 K min^–1^. Afterward, the obtained phosphors were cooled naturally to room temperature and ground once again for subsequent structural and spectroscopic studies.

### Methods

The X-ray diffraction (XRD) analysis of obtained powders was performed using a PANalytical X’Pert Pro diffractometer equipped with an Anton Paar TCU1000 N Temperature Control Unit using Ni-filtered Cu Kα radiation (*V* = 40 kV, *I* = 30 mA). Measurements were performed in the 2θ = 10–70° range with a 0.02626° step and 60 min measurement time.

Scanning electron microscopy (SEM) was used to verify the morphology of the samples and the distribution of its elements by EDS mapping. The FEI Nova NanoSEM 230 equipped with an EDAX Genesis XM4 energy dispersive spectrometer was used for measurements (*V* = 30 kV for SEM and *V* = 5 kV for EDS mapping). Samples were prepared by dispersing some amount of powder in a few drops of methanol. A drop of the resulting suspension was placed on the carbon stub and dried.

The spectroscopic analysis (emission/excitation spectra and luminescence kinetics) was performed with the use of an FLS1000 Fluorescence Spectrometer from Edinburgh Instruments, equipped with a 450 W xenon lamp and an R5509-72 photomultiplier tube from Hamamatsu with nitrogen-flow cooled housing as the detector. The temperature-dependent measurements were carried out using a THMS 600 heating–cooling stage from Linkam, which provides a temperature stability of 0.1 K and a set point resolution of 0.1 K. Before each measurement, the temperature was stabilized for 2 min to ensure reliable readouts.

The average luminescence lifetime (τ_avr_) was calculated using eq 1, based on double- exponential fit of the luminescence decay curves (eq 2):

1 $$\tau_{\mathrm{avr}}=\frac{A_{1}{\tau_{1}}^{2}+A_{2}{\tau_{2}}^{2}}{A_{1}\tau_{1}+A_{2}\tau_{2}}$$

2 $$I\left(t\right)=I_{0}+A_{1}\cdot \exp\left(-\frac{t}{\tau_{1}}\right)+A_{2}\cdot \exp\left(-\frac{t}{\tau_{2}}\right)$$

To determine the *S*_R_, the thermal dependencies of τ_avr_ and LIR were fitted using a dose–response curve (eq 1 in SI). The experimental values of *S*_R_ were calculated using the obtained fitting curves at each *T*_*i*_ temperature as follows:

3 $$S_{R}\left(T_{i}\right)=\frac{100\%}{\Omega }\frac{\Omega \left(T_{i}\right)-\Omega \left(T_{i-1}\right)}{T_{i}-T_{i-1}}$$

where Ω­(*T*_*i*_) represents the τ_avr_ or LIR at temperature *T*_*i*_.

Both Ba_2_LaNbO_6_ and Ca_2_LaNbO_6_ belong to the class of double perovskite compounds with the general formula A_2_BB′O_6_, where A usually represents elements from the *s* or *p*-blocks, while B and B′ are elements from the *d*- or *f-*blocks.^47–56^ These compounds crystallize in a monoclinic crystal structure but differ in their space groups: *C*2/*m* ^47^ for Ba_2_LaNbO_6_ and *P*21/*c* ^57^ for Ca_2_LaNbO_6_. The large ionic radii of both Ca^2+^ or Ba^2+^ ions result in the fact that they occupy cuboctahedrally coordinated sites. Therefore, these ions are coordinated by 12 O^2–^ ions (Figure 1a,b). On the other hand, La^3+^ and Nb^5+^ are octahedrally coordinated.^48–56,58–63^ XRD analysis confirmed that the synthesized samples contain a single expected phase, with no additional reflections indicating impurities, as compared to the ICSD No. 172403 and JCPDS No. 70-1157 reference patterns for Ba_2_LaNbO_6_ and Ca_2_LaNbO_6_, respectively (Figure 1c; see also Figure S1). For Ca_2_LaNbO_6_, the obtained reflections were compared to the analogous Ca_2_LaTaO_6_ monoclinic structure due to the unavailability of a suitable reference pattern in the crystallographic database. The matching reflections with the reference data also indicate the successful incorporation of Mn^4+^ ions into the Ba_2_LaNbO_6_ and Ca_2_LaNbO_6_ structures at a molar amount of 1%. Based on the analysis of the ionic radii it is expected that Mn^4+^ ions preferentially occupy crystallographic positions that provide 6-fold coordination with ligands.^32,64,65^ Detailed information about Nb^5+^–O^2–^ bond length and angles is given in Tables S1 and S2 (see also Figure S2). From the analysis of these data, it can be clearly seen that higher symmetry of the (NbO_6_)^7–^ polyhedral can be found for Ba_2_LaNbO_6_. However, the aliovalent substitution of two Mn^4+^ ions by La^3+^ and Nb^5+^ sites which allows for charge compensation can also be considered, as was discussed by Liu et al. for A_2_BB′O_6_ (where A = Sr^2+^, Ca^2+^; B, B′ = In^3+^, Sb^5+^, Sn^4+^) doped Fe^3+^ ions.^66^ It is especially important in the case of Ca_2_LaNbO_6_ of the *P*21/*c* space group where cations are ordered and hence Mn^4+^ may aliovalently substitute both sites. On the other hand, random distribution of cation sites in Ba_2_LaNbO_6_ of *C*2/*m* reduced the probability of this type of substitution.

**Figure 1:**
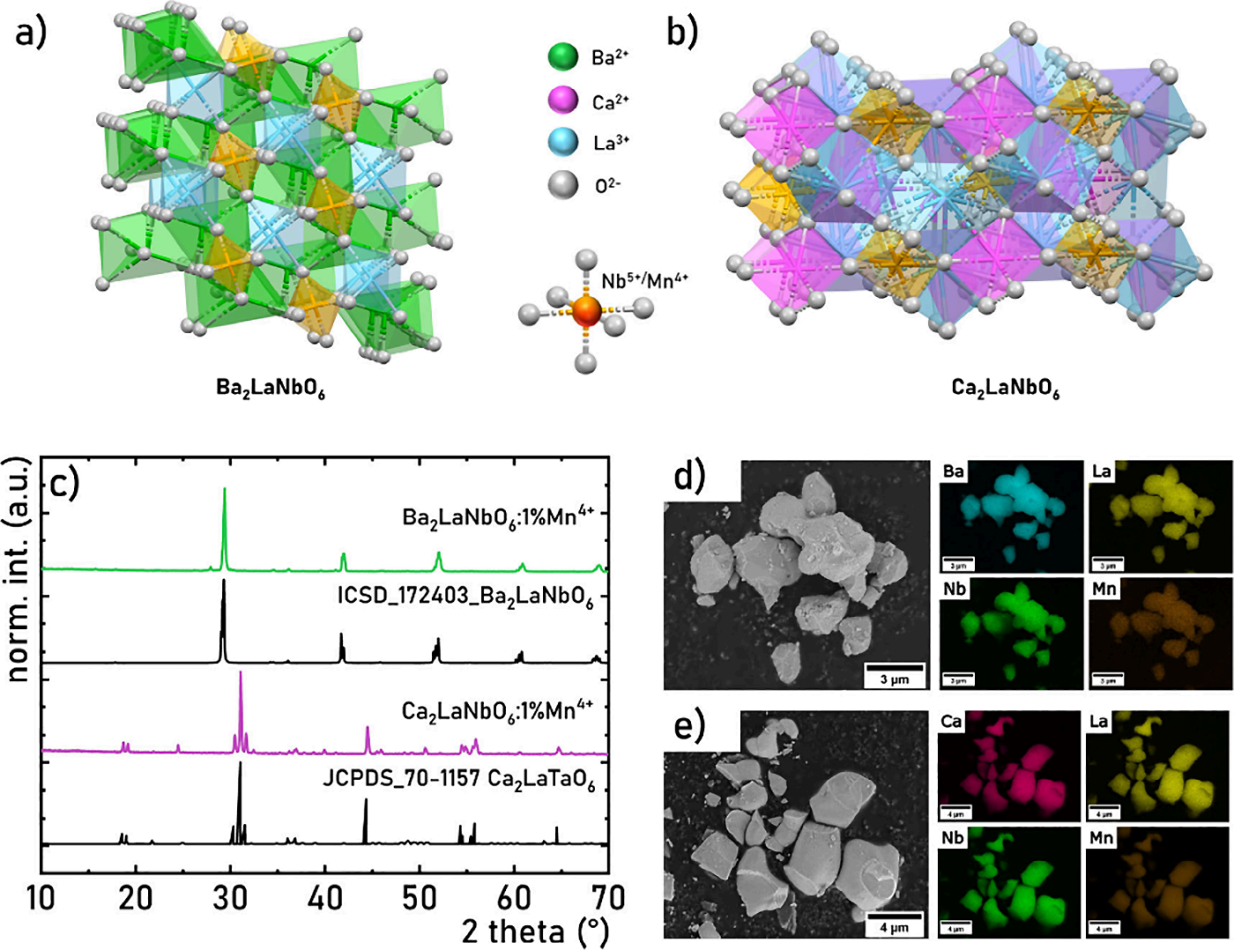
Visualization of Ba_2_LaNbO_6_ (a) and Ca_2_LaNbO_6_ (b) structures; comparison of the XRD patterns of Ca_2_LaNbO_6_:1%Mn^4+^ and Ca_2_LaNbO_6_:1%Mn^4+^ powders (c); representative SEM image and corresponding elemental maps of the Ba_2_LaNbO_6_:1%Mn^4+^ (d) and Ca_2_LaNbO_6_:1%Mn^4+^ (e).

The successful incorporation of a dopant ion into the host lattice is confirmed by the observed shift in the XRD reflections of the studied phosphors toward larger angles compared to their reference patterns. This shift indicates a reduction in the crystallographic unit cell size, associated with the substitution of smaller Mn^4+^ ions (0.53 Å) for Nb^5+^ ions, which have a larger ionic radius of 0.64 Å.^67^

SEM imaging revealed the presence of predominantly microcrystals in both Ba_2_LaNbO_6_ and Ca_2_LaNbO_6_:1%Mn^4+^ powders (Figure 1d,e, respectively). The crystals lack a uniform, characteristic shape and tend to aggregate, but their average size was estimated to be 3.0 ± 0.2 μm. The obtained morphology is a result of the high-temperature solid-state synthesis method used.

One of the key requirements for reliable spectroscopic characterization is the homogeneity of the samples in terms of elemental composition, especially dopant ions. Therefore, this feature was investigated using EDS analysis, which resulted in elemental distribution maps for Ba, Ca, La, Nb, and Mn. As shown in Figure 1d,e, both samples exhibit a high degree of elemental homogeneity, with an even distribution of all elements throughout the entire crystal studied. Furthermore, EDS analysis confirmed the successful incorporation of Mn^4+^ dopant ions into the host structures.

The luminescence of Mn^4+^ ions arises from the depopulation of the ^2^E level to the ^4^A_2_ ground state.^29,31–36,39,40^ The ^2^E → ^4^A_2_ electron transition is spin-forbidden and corresponds to photon emission in the red spectral range (Figure 2a). According to the Tanabe–Sugano diagram for transition metal ions with a *3d* ^3^ electron configuration, the energy of the ^2^E level is nearly independent of the crystal field strength.^68,69^ Therefore, variations in the crystal field strength acting on Mn^4+^ ions due to changes in the host material composition have a minimal impact on the spectral position of the ^2^E → ^4^A_2_ emission band. However, the energy of the ^2^E level depends on the covalency of the Mn^4+^–O^2–^ bond, leading to slight shifts in the spectral position of Mn^4+^ emission with changes in the host material. While the spectral position of the ^2^E → ^4^A_2_ band is not highly sensitive to changes in crystal field strength, variations in this parameter can influence the thermal stability of Mn^4+^ luminescence. The primary thermally induced depopulation mechanism of the ^2^E level involves crossover processes between the ^2^E and ^4^T_2_ state parabolas. According to the ligand field theory for perfect octahedral sited occupied by Mn^4+^ ions the energy of the ^4^T_2_ level increases with stronger *Dq*/*B* crystal fields, and *Dq*/*B* is proportional to *R* ^–5^ (where *R* is the Mn^4+^–O^2–^ distance). Therefore, materials with shorter Mn^4+^–O^2–^ distances are expected to exhibit higher thermal stability of luminescence.^35^ The energy of the ^4^T_2_ level also affects the luminescence kinetics of the ^2^E level. Spin–orbit coupling between the ^2^E and ^4^T_2_ levels partially relaxes the ^2^E → ^4^A_2_ electron transition, as the ^4^T_2_ → ^4^A_2_ transition is spin-allowed. Consequently, materials with stronger crystal fields exhibit a larger energy separation between the ^2^E and ^4^T_2_ levels, reducing spin–orbit coupling and prolonging the lifetime of the ^2^E state. However, when discussing thermal stability of the Mn^4+^ luminescence in the analyzed host materials additional thermally activated processes like ligand to metal charge transfer transition process (Nb^5+^ ← O^2–^ LMCT) should also be considered.^70^ A comparison of the luminescence spectra of Ba_2_LaNbO_6_:1%Mn^4+^ and Ca_2_LaNbO_6_:1%Mn^4+^ reveals notable differences in the spectroscopic properties of the Mn^4+^ ions. For Ba_2_LaNbO_6_:1%Mn^4+^, at 83 K, the zero-phonon line and vibrational lines associated with the ^2^E → ^4^A_2_ transition are distinctly observable (Figure 2b, Figure S3). In contrast, for Ca_2_LaNbO_6_:1%Mn^4+^, vibrational lines are not distinguishable in the spectrum, even at low temperatures and under low excitation power density, likely due to inhomogeneous broadening caused by localized distortions of the crystallographic sites occupied by Mn^4+^ ions.^54,58,60^ This effect can be explained by the aliovalent substitution of both cationic sites by Mn^4+^ ions in this compound. The higher energy of the ^2^E → ^4^A_2_ band for Ba_2_LaNbO_6_:1%Mn^4+^ (15,120 cm^–1^) compared to that of Ca_2_LaNbO_6_:1%Mn^4+^ (14,200 cm^–1^) suggests that the average Mn^4+^–O^2–^ distance is greater in Ba_2_LaNbO_6_:1%Mn^4+^. A comparison of the excitation spectra of Ba_2_LaNbO_6_:1%Mn^4+^ and Ca_2_LaNbO_6_:1%Mn^4+^, measured at 83 K, indicates that both spectra consist of five bands associated with ^4^A_2_ → ^4^T_2_ electronic transitions (20,062 cm^–1^ for Ba_2_LaNbO_6_:1%Mn^4+^ and 19,783 cm^–1^ for Ca_2_LaNbO_6_:1%Mn^4+^), ^4^A_2_ → ^2^T_2_ electronic transitions (26,921 cm^–1^ for Ba_2_LaNbO_6_:1%Mn^4+^ and 24,635 cm^–1^ for Ca_2_LaNbO_6_:1%Mn^4+^), ^4^A_2_ → ^4^T_1_ electronic transitions (29,180 cm^–1^ for Ba_2_LaNbO_6_:1%Mn^4+^ and 27,170 cm^–1^ for Ca_2_LaNbO_6_:1%Mn^4+^), Mn^4+^ → O^2–^ electronic transitions (31,684 cm^–1^ for Ba_2_LaNbO_6_:1%Mn^4+^ and 29,182 cm^–1^ for Ca_2_LaNbO_6_:1%Mn^4+^), and a group of electronic transitions from the ground ^4^A_2_ state to the closely located orbital triplets and doublets (all are split by low-symmetry crystal field) coming from the ^2^G, ^2^H, ^2^P terms of the Mn^4+^ ions (35,139 cm^–1^ for Ba_2_LaNbO_6_:1%Mn^4+^ and 32,170 cm^–1^ for Ca_2_LaNbO_6_:1%Mn^4+^) (Figure 2c, Figures S4 and S5). Higher values for the ^4^A_2_ → ^4^T_2_ and ^4^T_1_ band energies in Ba_2_LaNbO_6_:1%Mn^4+^ suggest a stronger crystal field acting on Mn^4+^ ions in this host material compared to Ca_2_LaNbO_6_:1%Mn^4+^. To verify this hypothesis, the crystal field strength (*Dq*/*B*) and Racah parameters (*B* and *C*) were determined using the following equations:^32,71^

4 E(A24→T24)=10Dq

5 $$\frac{Dq}{B}=\frac{15\left(\frac{\Delta E}{Dq}-8\right)}{{\left(\frac{\Delta E}{Dq}\right)}^{2}-10\frac{\Delta E}{Dq}}$$

6 E(E2→A24)B=3.05CB+7.9−1.8BDq

**Figure 2:**
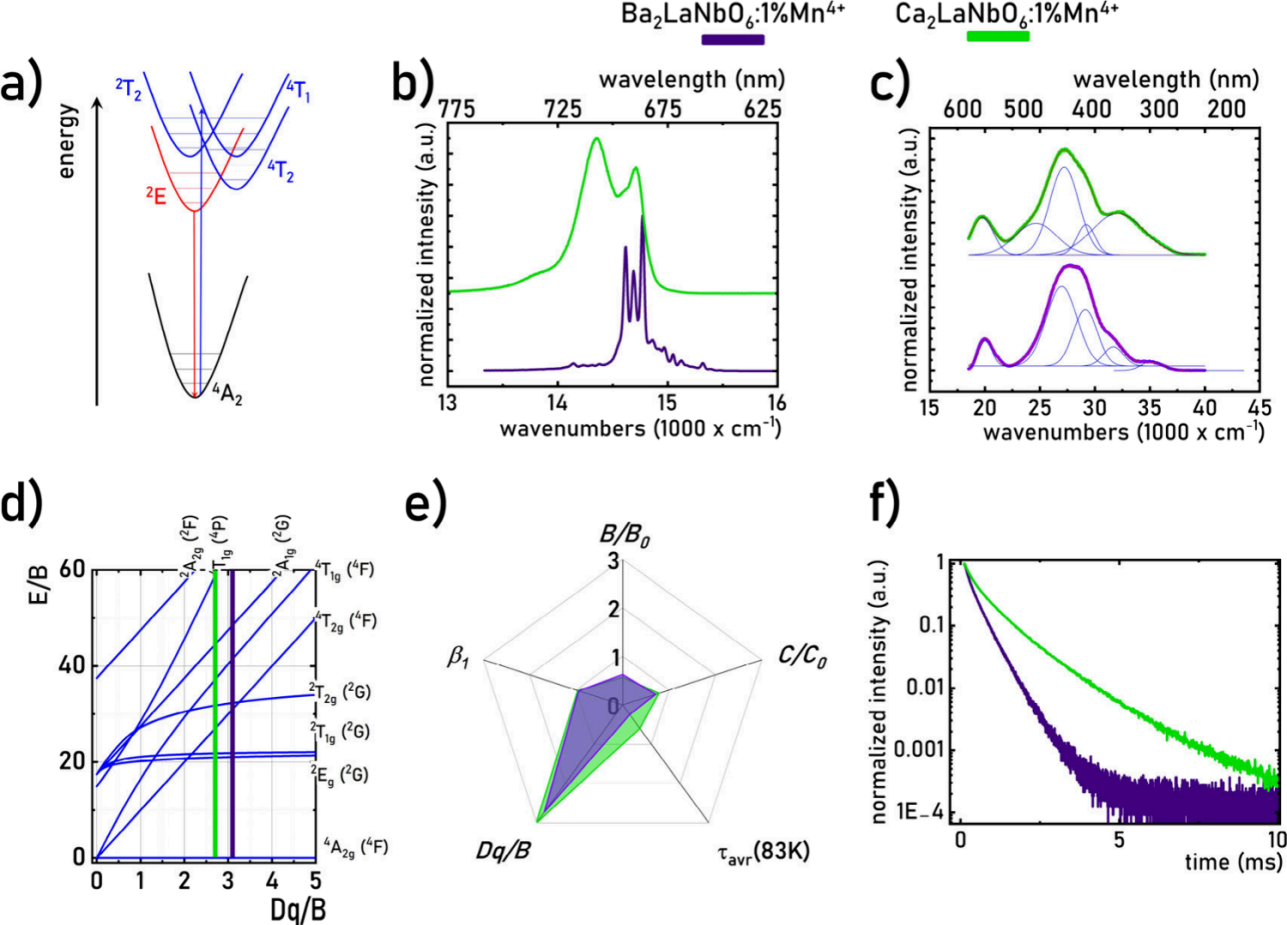
Simplified configurational-coordination diagram of Mn^4+^ ions (a) the comparison of emission (λ_exc_ = 359 nm for Ba_2_LaNbO_6_:1%Mn^4+^ and λ_exc_ = 367.5 nm for Ca_2_LaNbO_6_:1%Mn^4+^) (b) and excitation (λ_em_ = 680.75 nm for Ba_2_LaNbO_6_:1%Mn^4+^ and λ_em_ = 696.5 nm for Ca_2_LaNbO_6_:1%Mn^4+^) (c) spectra of Ba_2_LaNbO_6_:1%Mn^4+^ and Ca_2_LaNbO_6_:1%Mn^4+^ measured at 83 K; Tanabe-Sugano diagram for 3d^3^ electronic configuration (d); comparison of normalized Racah parameters *B*, *C*, β1, *Dq*/*B*, and τ_avr_ (at 83 K) for both phosphors (e) and the luminescence decay profiles obtained at 83K for analyzed phosphors (f).

The calculated values of *Dq*/*B*, *B*, and *C* were 3.02, 662, and 3370 for Ba_2_LaNbO_6_:1%Mn^4+^ and 2.73, 723, and 3068 for Ca_2_LaNbO_6_:1%Mn^4+^ (Figure 2d). It should also be noted here that since the aliovalent substitution of both cationic sites by Mn^4+^ ions is considered for Ca_2_LaNbO_6_:1%Mn^4+^, the crystal field parameters are of the average character. These results confirm the stronger crystal field interaction with Mn^4+^ ions in Ba_2_LaNbO_6_:1%Mn^4+^. Based on the Racah parameters, the covalency parameter was determined using the equation^72^

7 $$\beta_{1}=\sqrt{{\left(\frac{B}{B_{0}}\right)}^{2}+{\left(\frac{C}{C_{0}}\right)}^{2}}$$

where *B*_0_ and *C*_0_ are the values for free ions equal to 1160 and 4303 cm^–1^, respectively. The lower β_1_ = 0.9472 value for Ca_2_LaNbO_6_:1%Mn^4+^ indicates lower covalency than in the case of Ba_2_LaNbO_6_:1%Mn^4+^ (β_1_ = 0.9691), consistent with prior assumptions (Figure 2e). All these parameters are listed in Table 1.

**Table 1:** Crystal Field and Racah Parameters and Covalency Calculated for Ba_2_LaNbO_6_:Mn^4+^ and Ca_2_LaNbO_6_:Mn^4+^ Phosphors

**Parameter**	**Ba**_**2**_ **LaNbO**_**6**_ **:1%Mn** ^**4+**^	**Ca**_**2**_ **LaNbO**_**6**_ **:1%Mn** ^**4+**^
*Dq*/*B*	3.02	2.73
*B*	662	723
*C*	3370	3068
β_1_	0.969	0.9472

A comparison of luminescence decay profiles for the ^2^E level at 83 K shows slight deviations from the exponential behavior. To quantitatively analyze the luminescence decay, average lifetimes (τ_avr_) were calculated as described in Experimental Section (eqs 1 and 2). The τ_avr_ value for Ca_2_LaNbO_6_:1%Mn^4+^ is significantly longer than that for Ba_2_LaNbO_6_:1%Mn^4+^, consistent with the previously reported data (Figure 2f).^47,50–52,54,58,60–63^ However, longer τ_avr_ of ^2^E state of Mn^4+^ in Ca_2_LaNbO_6_:1%Mn^4+^ with respect to Ba_2_LaNbO_6_:1%Mn^4+^ is not correlated with the difference in the crystal field strength. In the case of the perfect octahedral sites occupied by Mn^4+^ ions the radiative lifetime is expected to shorten when the crystal field strength decreases due to the more efficient mixing of the wave functions of the ^2^E state with the ^4^T_2_ one.^32,73^ Longer τ_avr_ in Ca_2_LaNbO_6_:1%Mn^4+^ may suggest that additional processes are involved here. The most probable process in this case is the population of the ^2^E state from the defects states, which serves as an optical trap.^74,75^ The formation of defects is especially probable when the aliovalent substitution of two Mn^4+^ ions by La^3+^ and Nb^5+^ sites occurs. This effect correlates with the broadening of the emission band of Mn^4+^ ions in Ca_2_LaNbO_6_:1%Mn^4+^. While this hypothesis is plausible, additional research is needed to confirm it experimentally, which is beyond the scope of this work.

To examine the effect of temperature on the spectroscopic properties of the analyzed materials, luminescence spectra were measured in the 83–403 K range (Figure 3a,b). As is evident from the presented spectra, an increase in temperature within this range does not cause any shift in the spectral position of the ^2^E → ^4^A_2_ bands for both phosphors. This indicates the absence of thermally induced distortions at the crystallographic sites occupied by the Mn^4+^ ions. A comparison of the integrated luminescence intensities of Mn^4+^ ions in the two materials reveals a significant difference in the rate of thermal quenching (Figure 3c). For Ba_2_LaNbO_6_:1%Mn^4+^, an increase in temperature initially has no notable effect on the integrated emission intensity up to approximately 240 K. Above this temperature, a sharp decrease in the luminescence intensity is observed. In contrast, for Ca_2_LaNbO_6_:1%Mn^4+^, thermal quenching begins as early as 83 K, with *T*_50_ (the temperature at which the luminescence intensity drops to 50%) determined to be 263 K, compared to *T*_50_ = 322 K for Ba_2_LaNbO_6_:1%Mn^4+^. These results suggest that Ba_2_LaNbO_6_:1%Mn^4+^ exhibits a higher thermal stability of luminescence, which is attributed to a higher activation energy. Analysis of the luminescence decay profiles from the ^2^E level of Mn^4+^ ions shows a monotonic decrease in the average lifetime (τ_avr_) with increasing temperature for both host materials (Figure 3d,e and Figure S6; fitting results are shown in Figures S7–S24). In Ca_2_LaNbO_6_:1%Mn^4+^, τ_avr_ decreases from 0.6 ms at 83 K to 0.07 ms at 403 K, whereas in Ba_2_LaNbO_6_:1%Mn^4+^, τ_avr_ reduces more drastically from 0.25 to 0.003 ms over the same temperature range. The rate of thermal shortening of τ_avr_ can be quantified using thermal relative sensitivity (*S*_R_) as follows:

8 $$S_{R\left[\tau \right]}=\frac{1}{\tau_{\mathrm{avr}}}\frac{\Delta \tau_{\mathrm{avr}}}{\Delta T}\cdot 100\%$$

where Δτ_avr_ corresponds to the change of τ_avr_ obtained for Δ*T* change in temperature. The obtained results for Ba_2_LaNbO_6_:1%Mn^4+^ and Ca_2_LaNbO_6_:1%Mn^4+^ reveal that in both cases *S*_R_ rises with temperature with maximal values around 403 K reaching *S*_Rmax_ = 1.2% K^–1^ for Ca_2_LaNbO_6_:1%Mn^4+^ and *S*_Rmax_ = 4.2% K^–1^ for Ba_2_LaNbO_6_:1%Mn^4+^ (Figure 3f).

**Figure 3:**
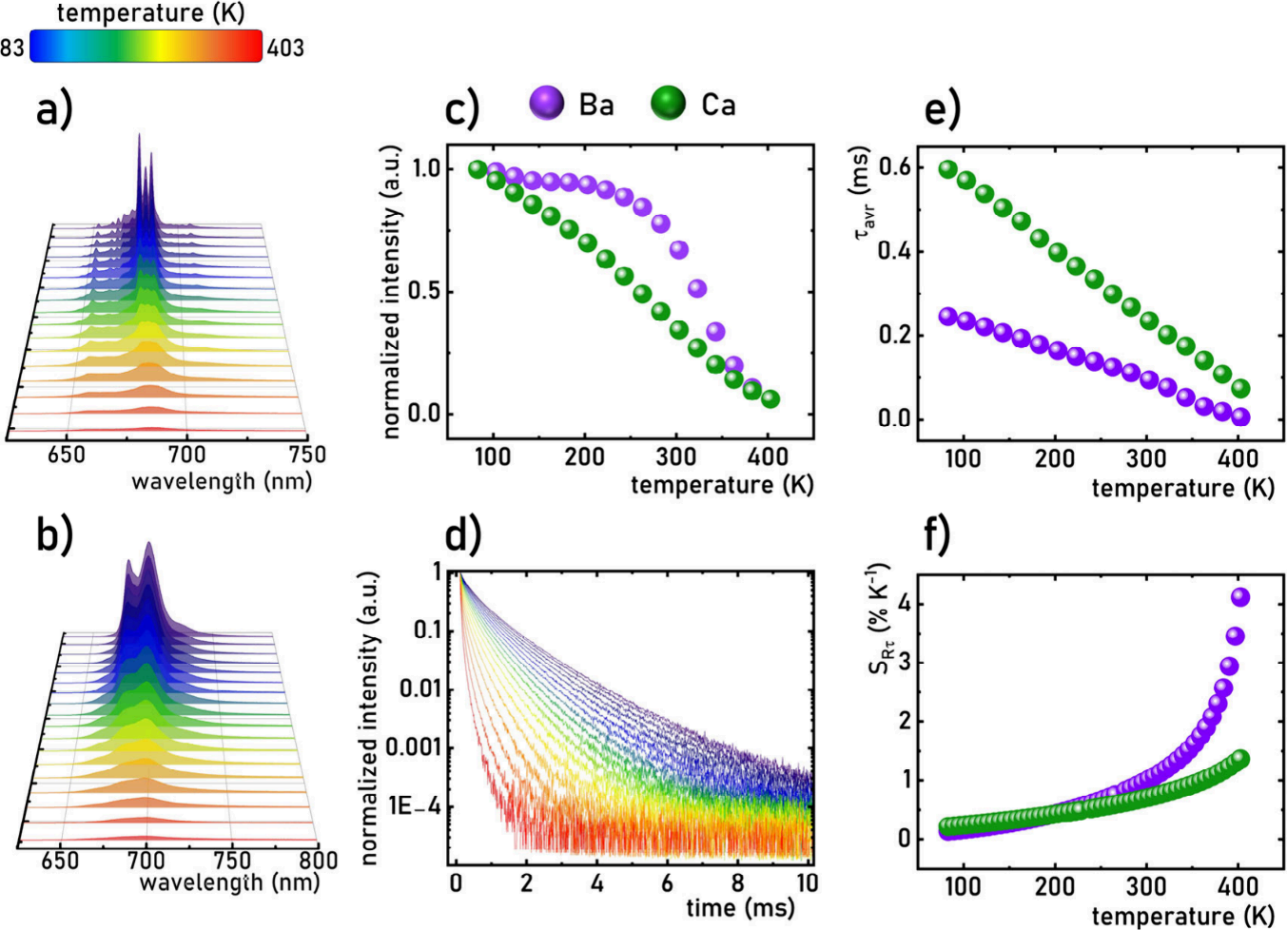
Emission spectra of Ba_2_LaNbO_6_:1%Mn^4+^ (a) and Ca_2_LaNbO_6_:1%Mn^4+^ (b) measured as a function of temperature (λ_exc_ = 359 nm); thermal dependence of normalized (to the intensity value obtained at 83 K) emission intensities of Ba_2_LaNbO_6_:1%Mn^4+^ and Ca_2_LaNbO_6_:1%Mn^4+^ (c); luminescence decay profiles for ^2^E → ^4^A_2_ electronic transition of Mn^4+^ ions in Ca_2_LaNbO_6_:1%Mn^4+^ (λ_exc_ = 367.5 nm, λ_em_= 696.5 nm) (d); and thermal dependence of average lifetimes of ^2^E state of Mn^4+^ ions in these host materials (e) and corresponding S_R_ (f).

The thermometric approach based on τ_avr_ requires measurement of the luminescence decay profile, which can be extremely time-consuming, particularly when two-dimensional imaging of the temperature distribution of an analyzed object is required. To address this limitation, a much simpler and faster solution—especially relevant for thermal imaging—is an approach based on the analysis of luminescence intensity recorded within two temporal gates. In this method, the thermometric parameter is defined as the ratio of luminescence intensities recorded in two time gates, and thermal distribution maps are synthesized by dividing images captured in these time windows. This approach is straightforward to implement because most cameras can adjust acquisition time, allowing gate durations to be modified without requiring complex methodologies. To verify the effectiveness of this approach, the integral luminescence intensity within different time gates of varying durations and delays relative to the excitation beam was calculated. The ^2^E → ^4^A_2_ emission intensity of Mn^4+^ ions was integrated over the following temporal ranges (indicated in Figure 4a): *A* = [0–0.1] ms, *B* = [0.1–0.2] ms, *C* = [0.2–0.3] ms, *D* = [0.3–0.4] ms, *E* = [0–0.5] ms, *F* = [0.5–1.0] ms, *G* = [0–1] ms, *H* = [1–2] ms, *I* = [0–2] ms, *J* = [2–4] ms, and *K* = [0–4] ms. The thermal variation of the emission intensity for each gate was analyzed for Ba_2_LaNbO_6_:1%Mn^4+^, as presented in Figure 4b. For shorter gates (e.g., *A*, *B*, *C*, *D*, *E* each ∼0.1 ms), an initial slight decrease in intensity with increasing temperature was observed until approximately 300 K, beyond which a sharp drop in intensity occurred. This behavior is attributed to the fact that the highest luminescence intensities are observed during the initial phase of the decay profile, regardless of its overall shape. As the gate is delayed further from the excitation pulse, the initial thermal intensity variation becomes more pronounced, while the decrease in the intensity beyond 300 K becomes less steep. The temperature at which this intensity reduction is observed correlates with the thermal dependence of the total luminescence intensity (Figure 3c). For gates longer than 0.5 ms, a different trend was observed. Here, the initial increase in temperature caused a sharp drop in integral intensity, with the *J* gate exhibiting a particularly dramatic decrease, reaching 20% of the initial intensity by 200 K. These differences in the thermal dependence of Mn^4+^ emission intensities recorded at different gates highlight the feasibility of using the ratio of these intensities to remotely measure temperature. Six distinct luminescence intensity ratios (LIRs) were proposed and defined as follows:

9 LIR1=AB=∫0ms0.1ms[E2→A24]dt∫0.1ms0.2ms[E2→A24]dt

10 LIR2=AE=∫0ms0.1ms[E2→A24]dt∫0.4ms0.5ms[E2→A24]dt

11 LIR3=AF=∫0ms0.1ms[E2→A24]dt∫0ms0.5ms[E2→A24]dt

12 LIR4=FG=∫0ms0.5ms[E2→A24]dt∫0.5ms1.0ms[E2→A24]dt

13 LIR5=HI=∫0ms1ms[E2→A24]dt∫1ms2ms[E2→A24]dt

14 LIR6=JK=∫0ms2ms[E2→A24]dt∫2ms4ms[E2→A24]dt

**Figure 4:**
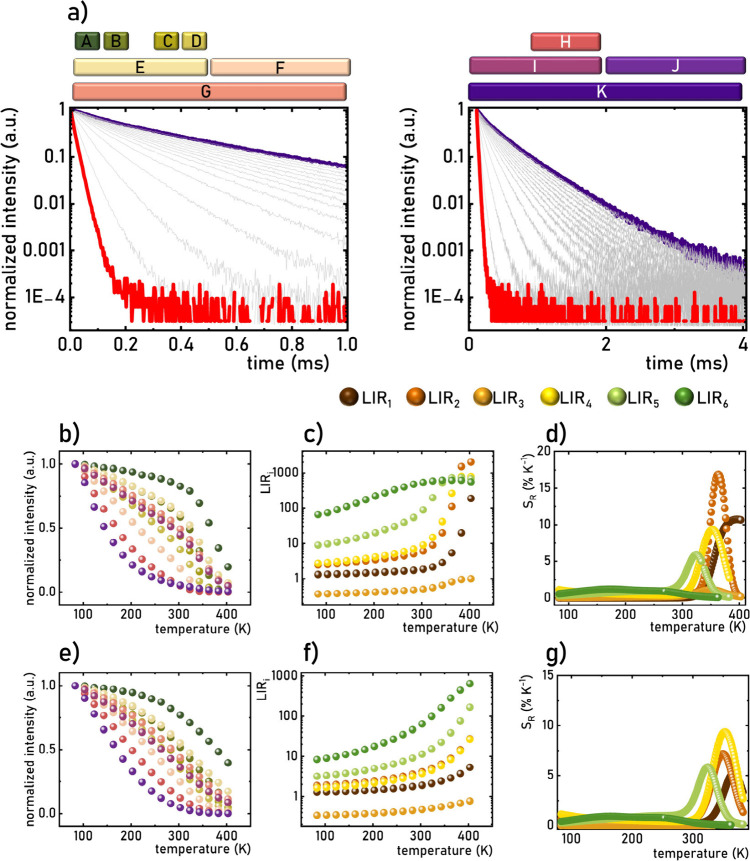
Representative luminescence decay profile of Mn^4+^ ions in Ba_2_LaNbO_6_:1%Mn^4+^ with marked temporal gates used in the analysis (a); the thermal dependence of emission intensity for Ba_2_LaNbO_6_:1%Mn^4+^ calculated in different temporal gates (b); LIR_*i*_ (c) and corresponding *S*_R_ (d); the thermal dependence of emission intensity for Ca_2_LaNbO_6_:1%Mn^4+^ calculated in different temporal gates (e); LIR_*i*_ (f) and corresponding *S*_R_ (g).

The design of the LIRs ensured an increase in their values across the entire analyzed temperature range (Figure 4c). For LIR_1_ to LIR_4_, temperature increases up to 300 K had minimal impact on their values; however, beyond 300 K, a sharp rise—reaching nearly 3 orders of magnitude for LIR_4_—was observed. Conversely, LIR_6_ displayed a continuous increase throughout the analyzed range, with saturation occurring above 310 K. Quantitative analysis of these thermal variations was performed by calculating the relative sensitivity using a method analogous to eq 8:

15 $$S_{R}=\frac{1}{\mathrm{LIR}}\frac{\Delta \mathrm{LIR}}{\Delta T}\cdot 100\%$$

The thermal variability of LIR_*i*_ was reflected in the *S*_R_ values, which peaked above 300 K (Figure 4d). The highest sensitivity, *S*_Rmax_ = 17.1% K^–1^, was recorded for LIR_2_ at 330 K, more than double the sensitivity achieved with the classical τ_avr_-based approach. Importantly, by selecting appropriate time gates and thus specific LIRs, the temperature corresponding to the maximum sensitivity can be adjusted. For example, *S*_Rmax_ values of 10.9% K^–1^, 1.4% K^–1^, 9.5% K^–1^, and 3.2% K^–1^ were obtained for LIR_1_, LIR_3_, LIR_4_, and LIR_5_, respectively. However, the thermal operating range for these parameters remained relatively narrow (∼60 K). Using LIR_6_, however, *S*_R_ > 1% K^–1^ was achieved across a broader range of 120–270 K. This flexibility is particularly important, as it allows the thermometric performance of the luminescence thermometer to be fine-tuned by adjusting the time gates to meet specific application requirements. Notably, this effect is not unique to Ba_2_LaNbO_6_:1%Mn^4+^; similar behavior and thermal variations in LIR_*i*_ were observed for Ca_2_LaNbO_6_:1%Mn^4+^, with maximum sensitivities of 5.2% K^–1^, 7.2% K^–1^, 0.9% K^–1^, 9.4% K^–1^, and 5.9% K^–1^ obtained for LIR_1_, LIR_2_, LIR_3_, LIR_4_, and LIR_5_, respectively (Figure 4e–g). It is very important to notice that the duration of the time gate affects the signal-to-noise ratio (SNR) (Figure S25), which affects the precision of the temperature readout. An increase of the duration of the time gates leads to an enhancement of SNR, reducing the influence of noise on temperature readouts. Since the emission intensity of Ba_2_LaNbO_6_:1%Mn^4+^ is higher with respect to Ca_2_LaNbO_6_:1%Mn^4+^, higher SNR is achieved for Ba_2_LaNbO_6_:1%Mn^4+^. Moreover, it should be underlined here that for both readout modes, the one based on the analysis of the luminescence decay profile and that based on the time-gates, the longer the integration time or the greater the number of measured repetitions of luminescence decay profiles, the higher the signal-to-noise ratio. It is also worth mentioning that the repeatability of the representative LIRs values during heating–cooling cycles was confirmed (Figure S26). It should be also considered here that the selection of LIR affects not only the maximal *S*_R_ and thermal operating range of the luminescence thermometers but also the temperature determination uncertainty (Figure S27). High *S*_R_ obtained for LIR_2_ for Ba_2_LaNbO_6_:1%Mn^4+^ allows determining temperature with *δT* as low as 0.1 K but only in a relatively narrow thermal range. On the other hand, using *S*_R_ much higher *δT* > 0.5 K but in a relatively wider thermal range. Therefore, selection of an LIR parameter used for temperature readout should be correlated with the requirements of a given application. The results presented in this study underscore the significant potential of this approach for remote temperature sensing. The advantages of high sensitivity, ease of implementation, and compatibility with thermal imaging warrant further exploration of this methodology. While the current study focuses on microcrystalline powders doped with Mn^4+^ ions, the discussed technique is equally applicable to nanocrystals. However, in such cases, additional factors must be considered, including energy diffusion processes, scattering phenomena, effective refractive index variations, and other photonic effects that can influence the luminescence kinetics of nanocrystals.^76,77^ Moreover, in systems codoped with multiple optical ions, the population pathways of the emitting levels—particularly the lifetimes of both the emitting and optically pumped levels—can alter the luminescence behavior.^76,78^ Considering that the duration of the excitation pulse can also impact the observed luminescence kinetics in these systems, it is often more advantageous to utilize phosphors doped with a single type of ion for such applications. Obviously, the further modification of the gate duration and change in the delay between gates allows an infinite number of other LIRs that can be obtained, with different corresponding *S*_R_ values. The primary objective of our investigation into the influence of gate duration and temporal spacing on the thermal dependence of LIR, and consequently on relative sensitivity, is to demonstrate a means of tuning both the thermal operating range and sensitivity of the thermometer without altering the material system itself. Importantly, short time gates capturing the early phase of the luminescence decay are advantageous in achieving high relative sensitivity and thermal resolution, primarily due to the favorable signal-to-noise ratio in this temporal region. However, reducing the gate width necessitates the use of faster detection optics, which may present practical limitations in certain measurement systems. Although the physical principle of the proposed approach is fundamentally the same as that responsible for the shortening of the average luminescence lifetime of the ^2^E state of Mn^4+^ ions, namely, the increase in nonradiative depopulation probability of the excited state with rising temperature, the use of time gates instead of the analysis of the luminescence decay curves opens the possibility for fast thermal imaging.

The effectiveness of the proposed approach has been validated in this study using two representative materials from the double perovskite family. However, given the simplicity and versatility of the temperature readout method, it can be anticipated that this strategy may be broadly applicable to other phosphor systems. To substantiate this assumption, further investigations should be carried out using well-established Mn^4+^-doped phosphors, such as K_2_SiF_6_:Mn^4+^,^79,80^ to confirm the generalizability of the approach. Although the primary aim of this study is to demonstrate the advantages of the LIR-based approach utilizing two distinct time gates, it is important to emphasize that further investigation is required to comprehensively assess the accuracy of the temperature readout prior to its implementation in practical applications.

A comparison of the thermometric performance of various lifetime-based luminescence thermometers, as presented in Table 2, clearly demonstrates that the relative sensitivity achieved for the materials investigated in this study using the conventional lifetime-based approach is notably high. While some reports indicate even higher *S*_R_ values, such as 6.35% K^–1^ for Lu_3_Al_5_O_12_:Mn^4+^,^81^ the sensitivity values obtained using the time-gated mode for Ba_2_LaNbO_6_:1%Mn^4+^ surpass those previously reported for thermometers based on luminescence kinetics. This finding strongly supports the potential of the proposed time-gated approach as a promising strategy for thermal sensing. Nevertheless, it is important to note that employing this type of ratiometric methodology may result in a narrower thermal operating range for the thermometer, which could limit its applicability in certain scenarios.

**Table 2:** Comparison of the Thermal Performance of Different Lifetime-Based Luminescence Thermometers

**Phosphor**	**Thermal operating range [K]**	***S*** **_Rmax_** **[% K** ^**‑1**^ **]**	**T@SRmax [K]**	**ref**
Gd_2_ZnTiO_6_:Mn^4+^	300–400	0.34	483	^82^
Al_2_Mo_3_O_12_:Er^3+^/Yb^3+^	300–600	0.28	600	^83^
Sr_2_LuSbO_6_:Mn^4+^	40–550	0.286	550	^84^
LiTaO_3_:Ti^4+^,Eu^3+^	303–443	3.395	303	^85^
Bi_2_Ga_4_O_9_ Cr^3+^	300–550	3.26	550	^86^
ZnGa_2_O4:Cr^3+^	298–563	0.58	420	^87^
Y_3_Al_5_O_12_:Cr^3+^	123–573	0.55	384	^44^
Lu_3_Al_5_O_12_:Mn^4+^	307–383 K	6.35	345	^81^
MgTiO_3_:Mn^4+^	198–323	4.1	277	^81^
BaLaCaSbO_6_:Mn^4+^	77–503	1.09	503	^25^
Ba_2_LaNbO_6_:1%Mn^4+^ (lifetime mode)	80–403	4.2	403	This work
Ba_2_LaNbO_6_:1%Mn^4+^ (time-gates mode)	300–400	17.1	330	This work
Ca_2_LaNbO_6_:1%Mn^4+^ (lifetime mode)	80–403	1.2	403	This work
Ca_2_LaNbO_6_:1%Mn^4+^ (time-gates mode)	300–400	9.4	350	This work

In conclusion, this Letter introduces a novel methodology for determining temperature in lifetime-based luminescence thermometry by utilizing the ratio of luminescence intensities recorded at two distinct time gates. The effect of the gate duration on the thermometric performance of the luminescence thermometers was investigated using two representative phosphors doped with Mn^4+^ ions, differing in crystal field strength: Ba_2_LaNbO_6_:1%Mn^4+^ and Ca_2_LaNbO_6_:1%Mn^4+^. The study revealed that differences in the activation energy for thermal depopulation of the emitting ^2^E state influenced the rate of thermal quenching of the ^2^E → ^4^A_2_ emission and the associated luminescence kinetics. Specifically, the higher crystal field strength (*Dq*) in Ba_2_LaNbO_6_:1%Mn^4+^ resulted in a stable luminescence intensity up to approximately 240 K, beyond which thermal quenching became significant. In contrast, Ca_2_LaNbO_6_:1%Mn^4+^ exhibited a monotonic decrease in luminescence intensity across the entire temperature range analyzed. Thermal quenching of the ^2^E state lifetime was observed, with values decreasing from 0.25 to 0.003 ms for Ba_2_LaNbO_6_:1%Mn^4+^ and from 0.6 to 0.07 ms for Ca_2_LaNbO_6_:1%Mn^4+^ within the 83–403 K range. The resulting relative sensitivity (*S*_R_) of luminescence thermometers based on τ_avr_ was determined to be 4.2% K^–1^ and 1.2% K^–1^ for Ba_2_LaNbO_6_:1%Mn^4+^ and Ca_2_LaNbO_6_:1%Mn^4+^, respectively. However, as discussed, the classical approach, relying on measuring the luminescence decay profile, hinders thermal imaging due to the need for time-consuming point-by-point measurements. The proposed method, based on the ratio of luminescence intensities, offers a significant advantage by enabling rapid thermal imaging. Furthermore, this approach achieves considerably higher relative sensitivity *S*_Rmax_, reaching 17.1% K^–1^ and 9.4% K^–1^ for Ba_2_LaNbO_6_:1%Mn^4+^ and Ca_2_LaNbO_6_:1%Mn^4+^, respectively. Importantly, adjusting the duration of the time gates allows the modulation of the thermometric performance. Short time gates corresponding to the initial luminescence decay phase yield high relative sensitivities but with a limited thermal operating range due to the pronounced intensity variations at this stage. Conversely, longer time gates (1–2 ms) provide significantly lower relative sensitivities but offer a much broader thermal operating range. The advantages of the proposed method, including the high relative sensitivity and suitability for fast thermal imaging, are expected to contribute to the widespread popularity of this approach in luminescence thermometry.

## Supplementary Material



## Notes

Emission and excitation spectra of Ba_2_LaNbO_6_:1%Mn^4+^ and Ca_2_LaNbO_6_:1%Mn^4+^ with their deconvolution, luminescence decay profiles of these phosphors with fitting curves, and signal-to-noise ratio (SNR) for the emission intensities of Ba_2_LaNbO_6_:1%Mn^4+^ and Ca_2_LaNbO_6_:1%Mn^4+^ integrated at different time gates calculated at 83 K (PDF)
