# Supplementary material for: Luminescence Thermometry Based on Time Gates: Highly Sensitive Approach for Real-Time Sensing and Imaging
Source: J Phys Chem Lett. 2025 Jun 6;16(24):5960–70. doi: 10.1021/acs.jpclett.5c01265 (PMC12183736; doi:10.1021/acs.jpclett.5c01265)
Supplement: Supplementary file 1 [file jz5c01265_si_001.pdf]

Supporting Information

Luminescence thermometry based on time gates: highly sensitive approach for real time sensing and imaging

M. Szymczak[1], D. Szymański[1], M. Piasecki[2], M. Brik[2,3,4,5,6], L. Marciniak[1*]

[1] Institute of Low Temperature and Structure Research, Polish Academy of Sciences,

Okólna 2, 50-422 Wrocław, Poland

[2] Faculty of Science and Technology, Jan Długosz University, Armii Krajowej 13/15, 42-200 Częstochowa, Poland

[3] School of Optoelectronic Engineering, Chongqing University of Posts and Telecommunications, Chongqing 400065, China

[4] Centre of Excellence for Photoconversion, Vinča Institute of Nuclear Sciences - National Institute of the Republic of Serbia, University of Belgrade, Belgrade, Serbia

[5] Institute of Physics, University of Tartu, W. Ostwald Str. 1, 50411 Tartu, Estonia

[6] Academy of Romanian Scientists, Ilfov Str. No. 3, 050044 Bucharest, Romania

*corresponding author: l.marciniak@intibs.pl

KEYWORDS: luminescence thermometry, optical sensors, lifetime-basded approach, thermal imaging, temporal rates

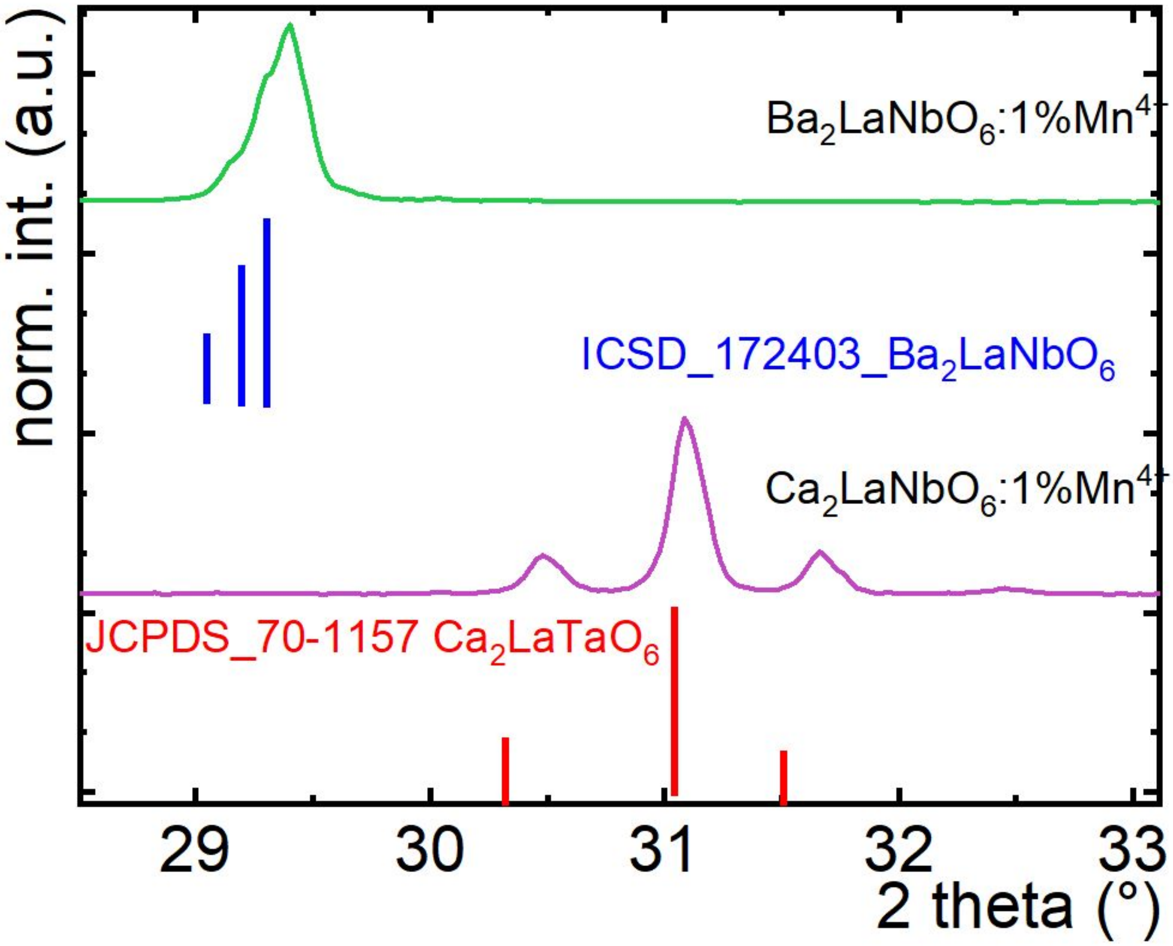

Figure S1. Comparison of the XRD patterns of Ba_2LaNbO_6:1%Mn^{4+} and Ca_2LaNbO_6:1%Mn^{4+} in the 28-33° 2theta range with the reference patterns.

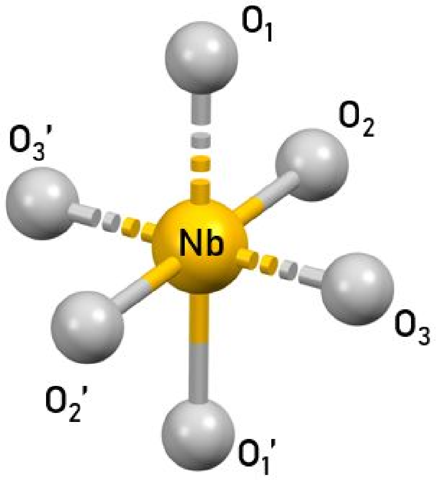

Figure S2. Schematic presentation of the octahedra of Nb and assignment of O ions used in Tables S1 and S2.

Table S1. The Nd-O bonds length in Ba_2LaNbO_6 and Ca_2LaNbO_6.

	**Ba_2LaNbO_6**	**Ca_2LaNbO_6**
**bond**	**length [A]**	**length [A]**
Nb-O_1	2.075	1.9817
Nb-O_1’	2.075	1.9817
Nb-O_2	2.075	2.0004
Nb-O_2’	2.075	2.0004
Nb-O_3	1.900	2.0449
Nb-O_3’	1.900	2.0449

Table S2. The angles between Nd-O bonds in Ba_2LaNbO_6 and Ca_2LaNbO_6.

	**Ba_2LaNbO_6**	**Ca_2LaNbO_6**
**atoms**	**angle [^{o}]**	**angle [^{o}]**
O_1-O_1’	180	180
O_1-O_2	81.83	45.079
O_1-O_2’	98.17	134.921
O_1-O_3	93.31	93.842
O_1-O_3’	86.69	86.158
O_2-O_2’	180	180
O_2-O_3	86.69	66.972
O_2-O_3’	93.31	113.028
O_2’-O_3	93.31	113.028
O_2’-O_3’	86.69	66.972
O_3-O_3’	180	180

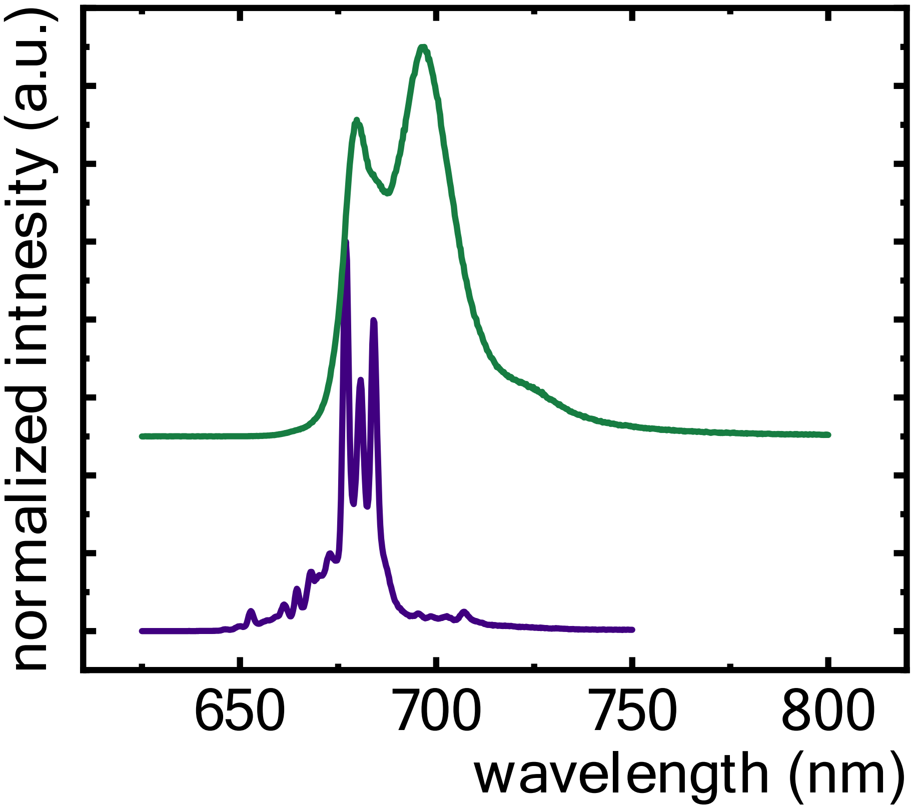

Figure S3.Normalized emission spectra of Ba_2LaNbO_6:1%Mn^{4+} (λ_{exc}=359 nm) and Ca_2LaNbO_6:1%Mn^{4+} (λ_{exc}=367.5 nm) measured at 83K.

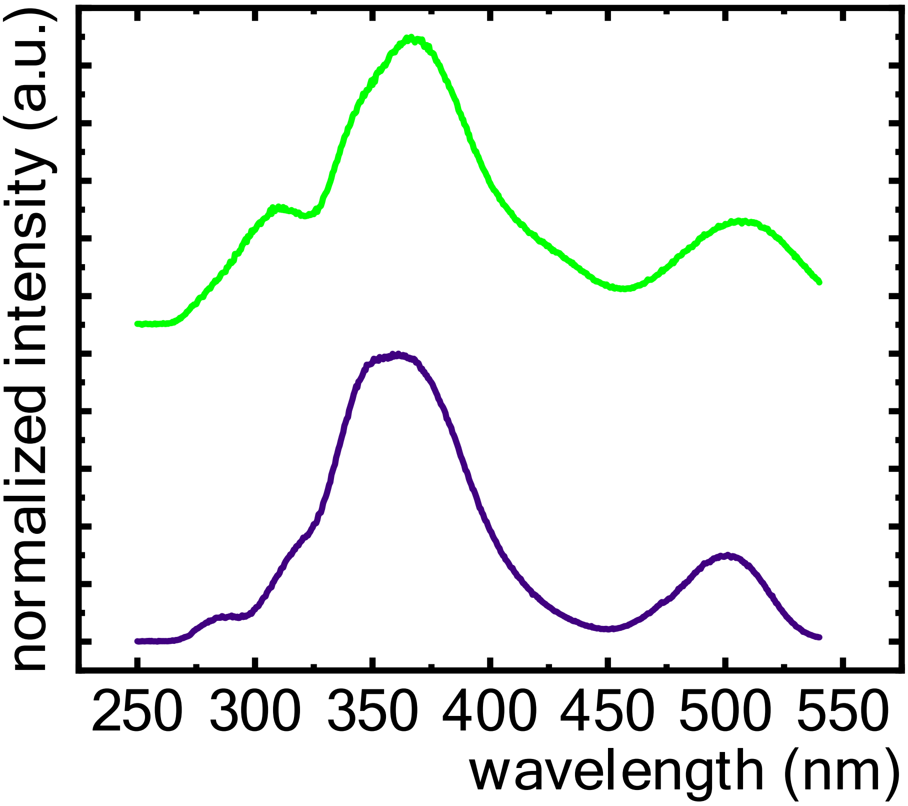

Figure S4.Normalized excitation spectra of Ba_2LaNbO_6:1%Mn^{4+} (λ_{em}=680.75 nm) (violet curve) and Ca_2LaNbO_6:1%Mn^{4+} (λ_{em}=696.5 nm) (green curve) measured at 83K.

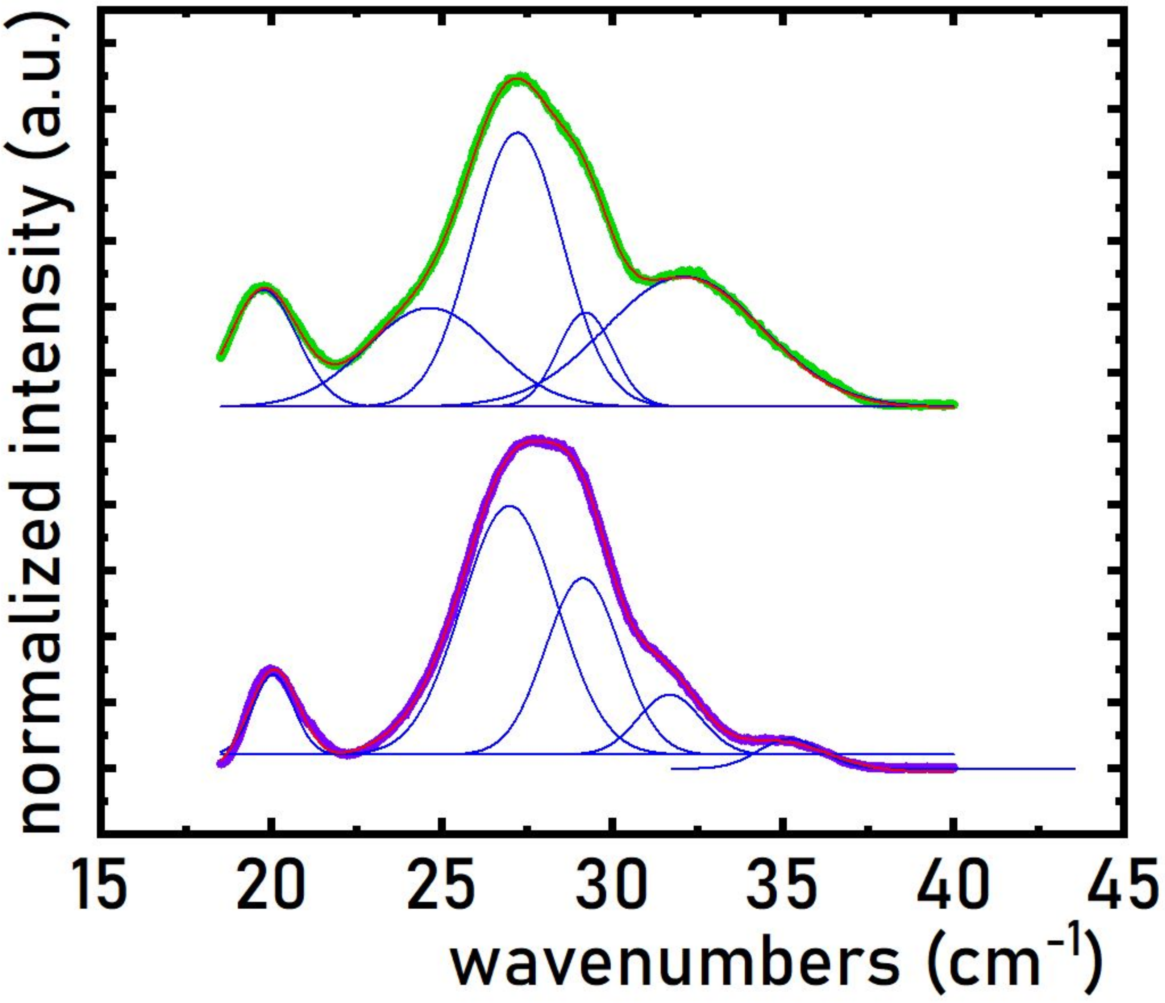

Figure S5. The results of the deconvolution of the excitation spectra of Ba_2LaNbO_6:1%Mn^{4+} (λ_{em}=680.75 nm) (violet curve) and Ca_2LaNbO_6:1%Mn^{4+} (λ_{em}=696.5 nm) (green curve) measured at 83K. Red line representative resulting curve of the deconvolution.

Thermal dependence of τ_{avr} and LIR were fitted using the following formula:

$$\Omega(T) = A_1 + \frac{A_2 - A_1}{1 + 10^{(\log x) - T)p}} \qquad \text{(S1)}$$

where Ω is the τ_{avr} or LIR.

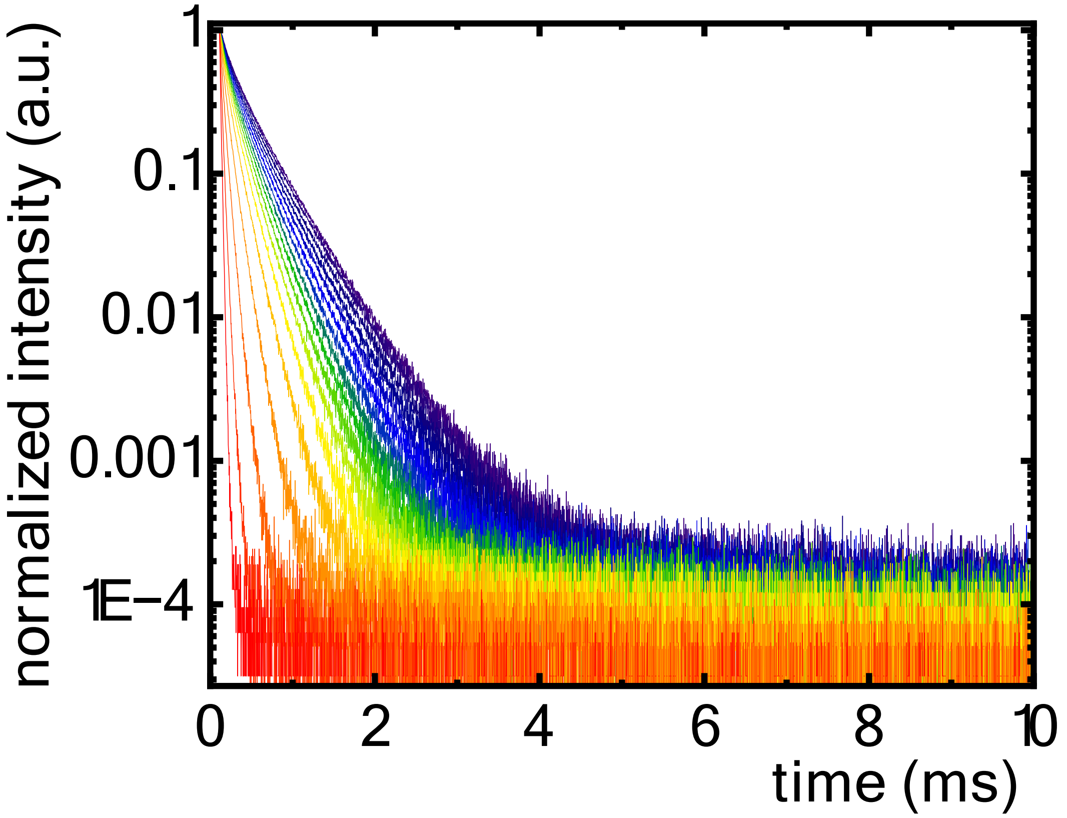

Figure S6.Normalized luminescence decay profiles of Ba_2LaNbO_6:1%Mn^{4+} (λ_{exc}=359 nm, λ_{em}=680.75 nm) measured as a function of temperature.

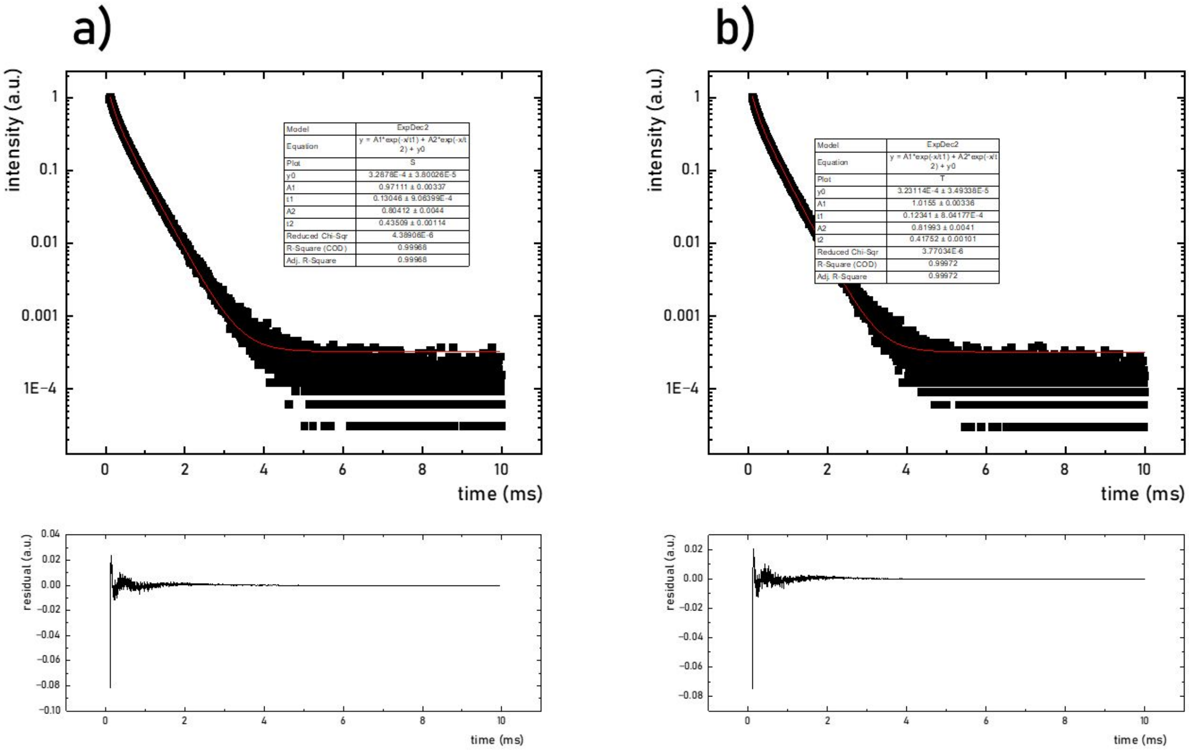

Figure S7. Luminescence decay with fitting curves for Ba_2LaNbO_6:1%Mn^{4+} (λ_{exc}=359 nm, λ_{em}=680.75 nm) measured at 83 K -a) and 103 K – b).

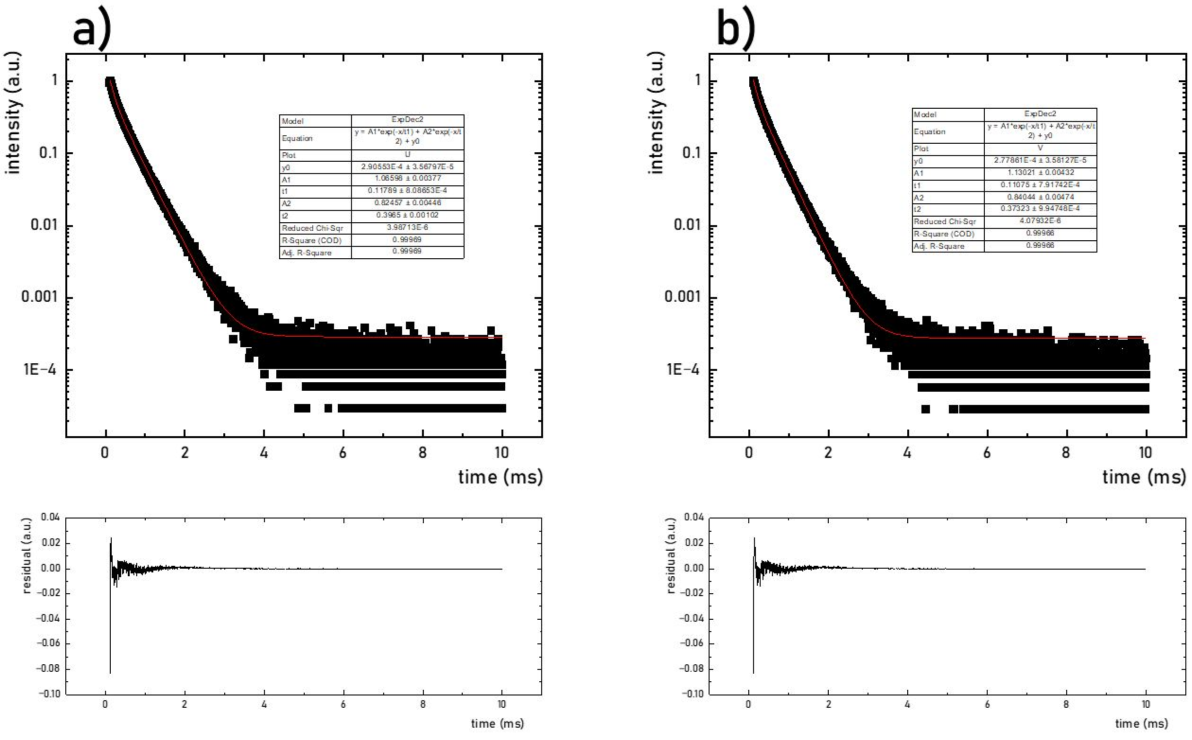

Figure S8. Luminescence decay with fitting curves for Ba_2LaNbO_6:1%Mn^{4+} (λ_{exc}=359 nm, λ_{em}=680.75 nm) measured at 123 K -a) and 143 K – b).

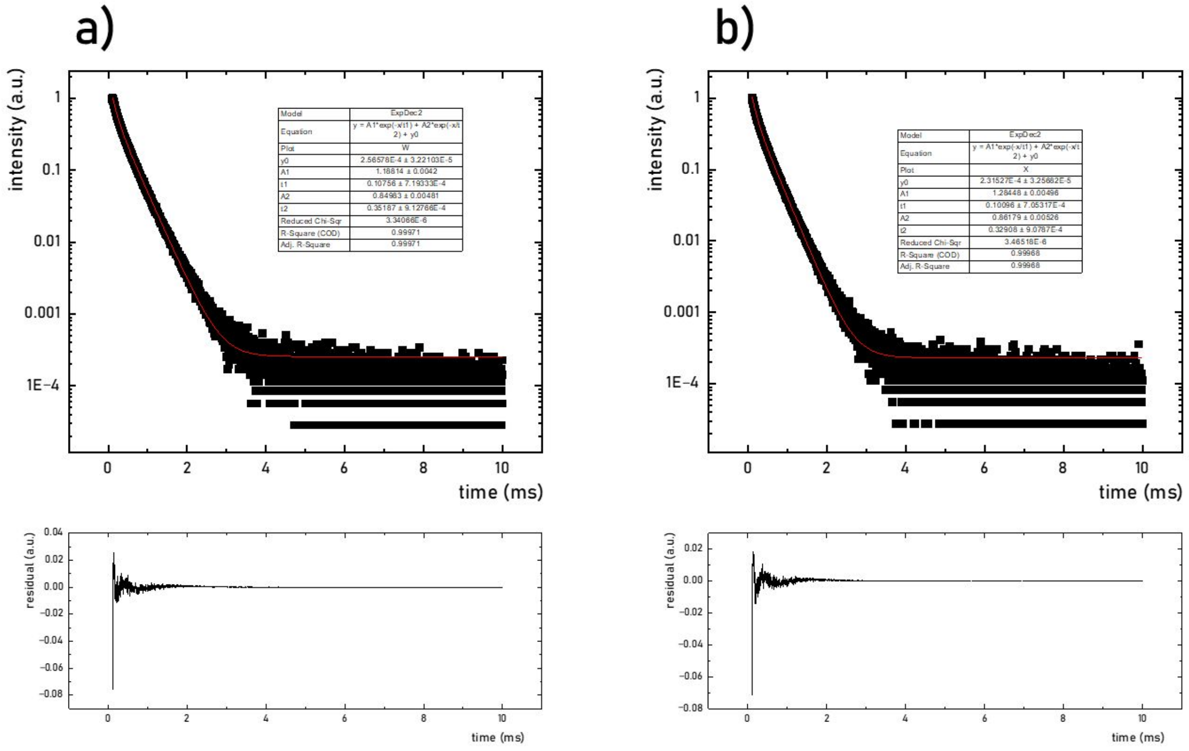

Figure S9. Luminescence decay with fitting curves for Ba_2LaNbO_6:1%Mn^{4+} (λ_{exc}=359 nm, λ_{em}=680.75 nm) measured at 163 K -a) and 183 K – b).

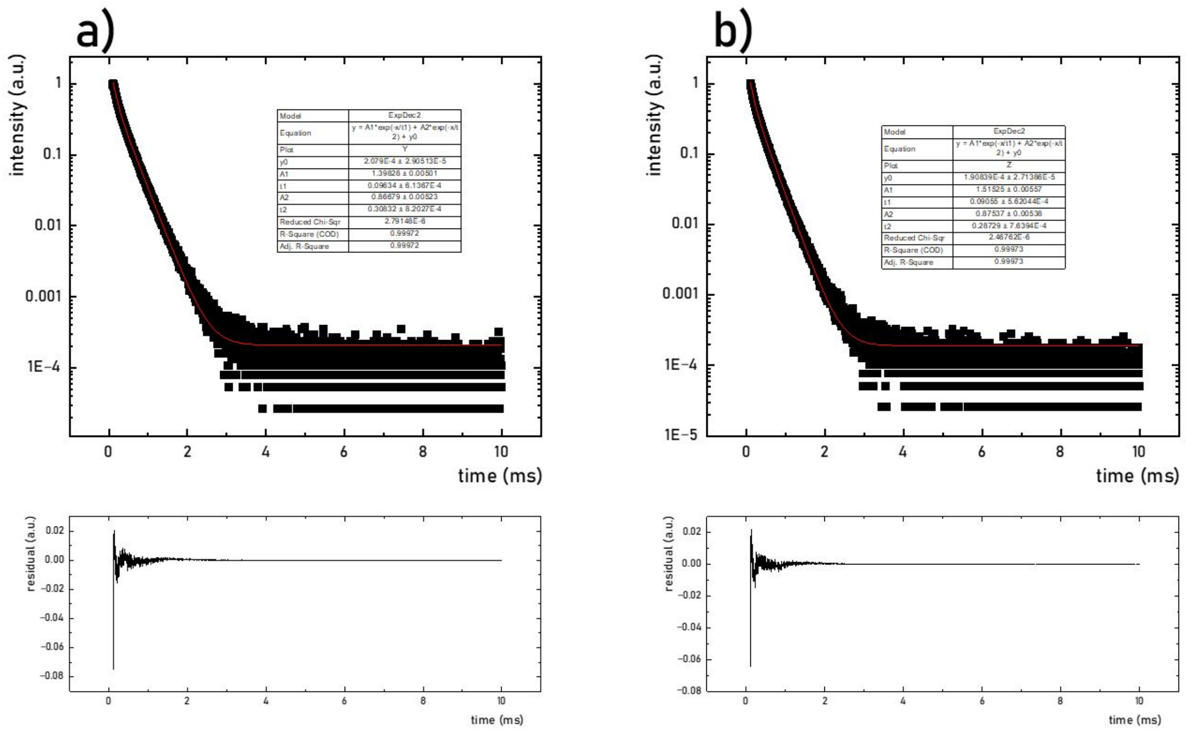

Figure S10. Luminescence decay with fitting curves for Ba_2LaNbO_6:1%Mn^{4+} (λ_{exc}=359 nm, λ_{em}=680.75 nm) measured at 203 K -a) and 223 K – b).

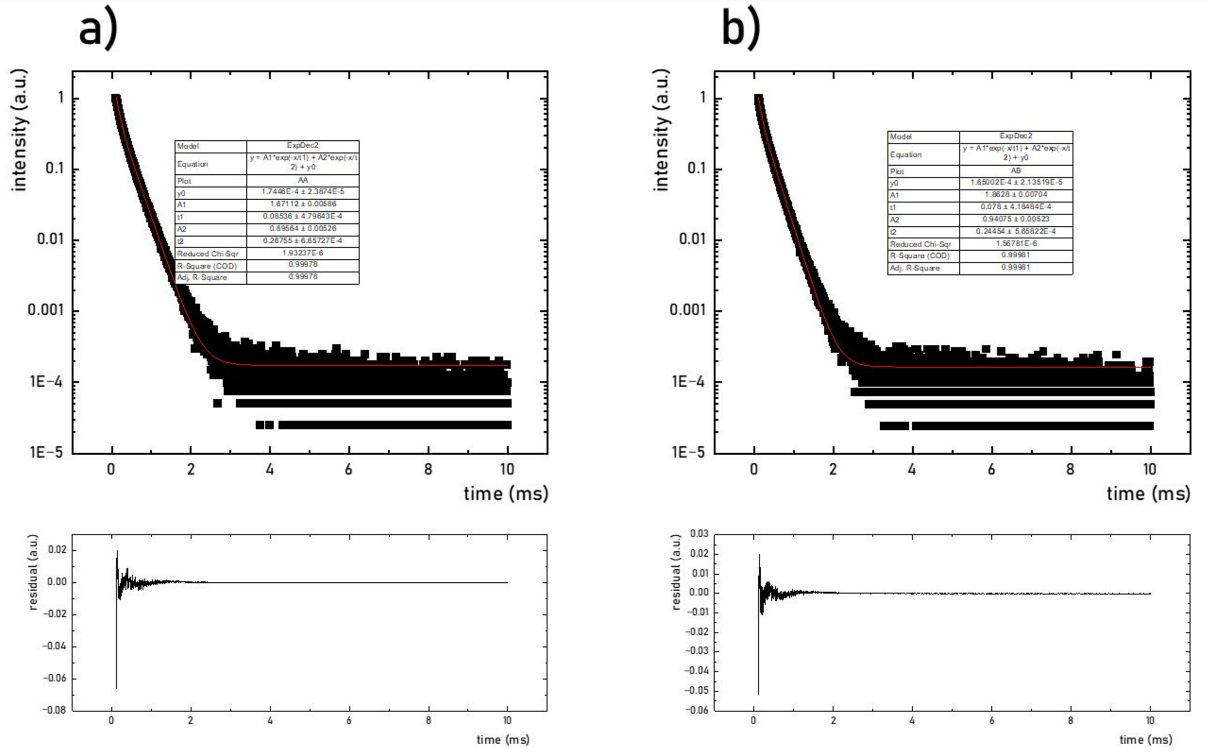

Figure S11. Luminescence decay with fitting curves for Ba_2LaNbO_6:1%Mn^{4+} (λ_{exc}=359 nm, λ_{em}=680.75 nm) measured at 243 K -a) and 263 K – b).

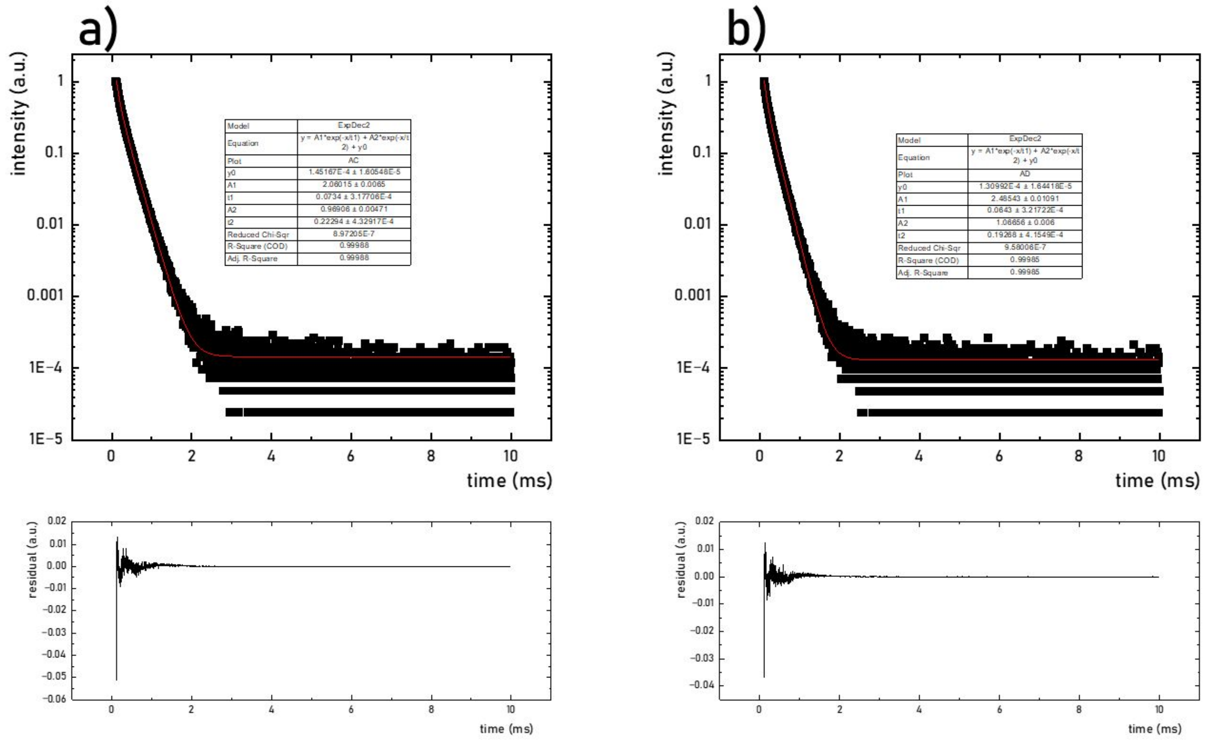

Figure S12. Luminescence decay with fitting curves for Ba_2LaNbO_6:1%Mn^{4+} (λ_{exc}=359 nm, λ_{em}=680.75 nm) measured at 283 K -a) and 303 K – b).

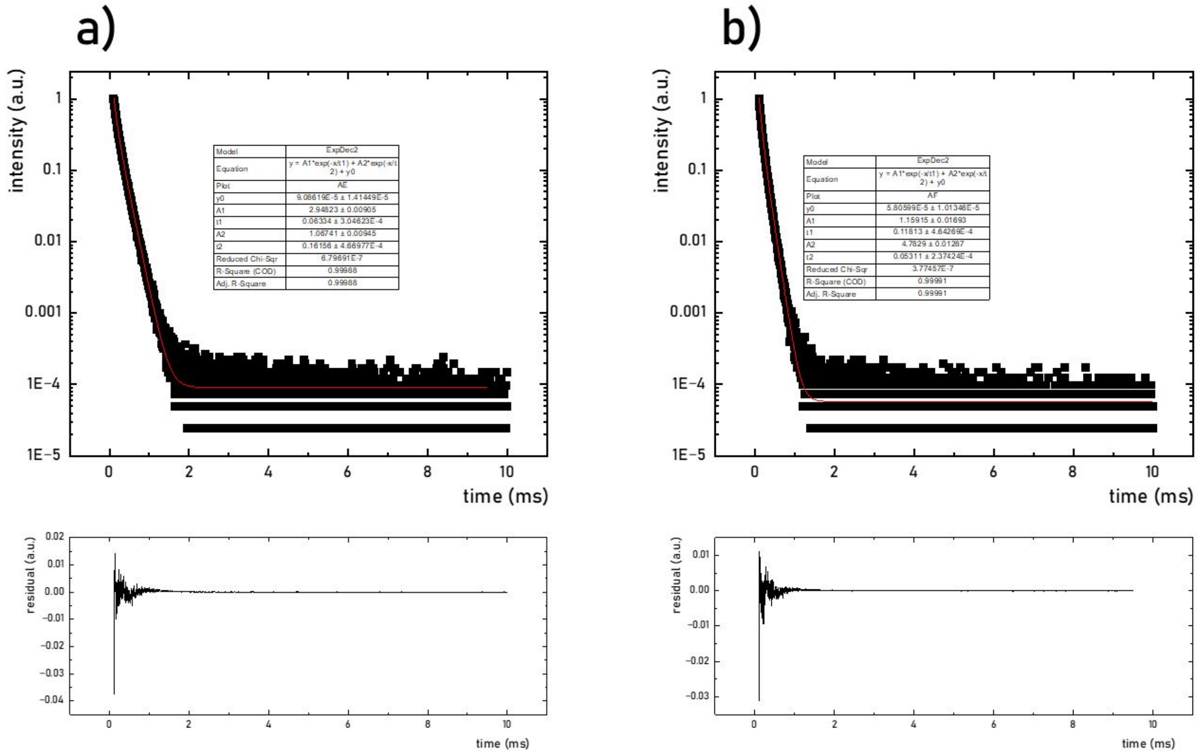

Figure S13. Luminescence decay with fitting curves for Ba_2LaNbO_6:1%Mn^{4+} (λ_{exc}=359 nm, λ_{em}=680.75 nm) measured at 323 K -a) and 343 K – b).

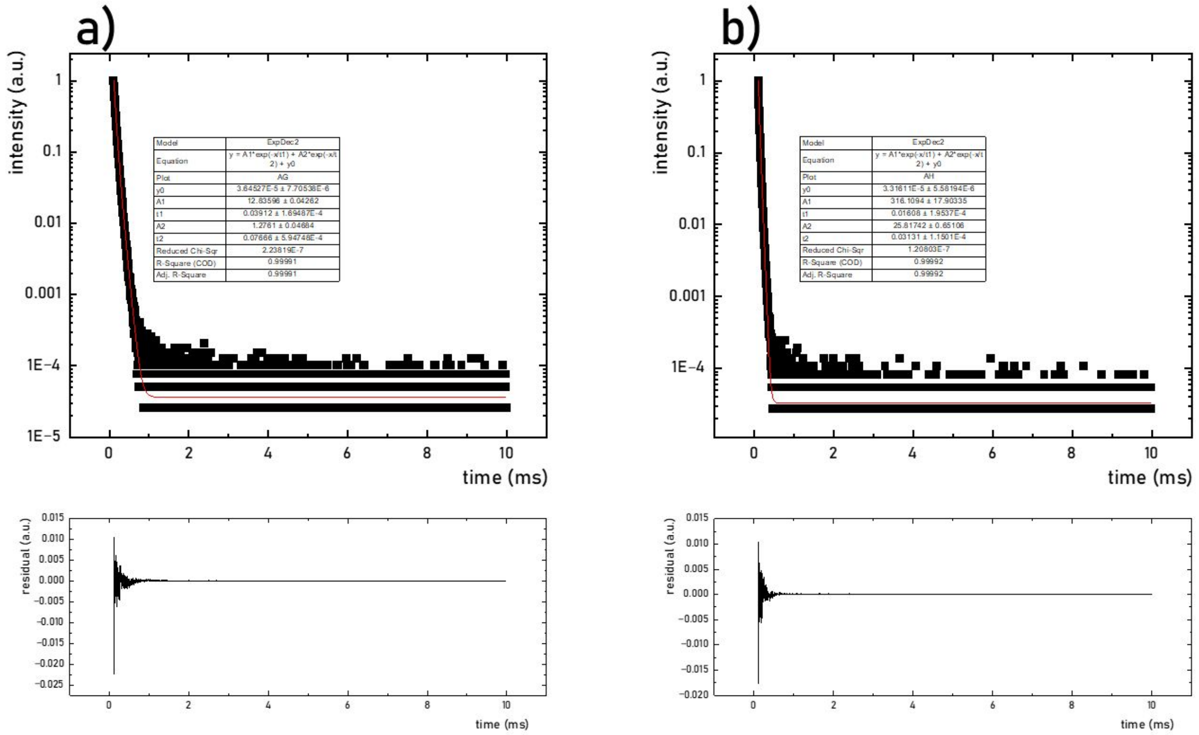

Figure S14. Luminescence decay with fitting curves for Ba_2LaNbO_6:1%Mn^{4+} (λ_{exc}=359 nm, λ_{em}=680.75 nm) measured at 363 K -a) and 383 K – b).

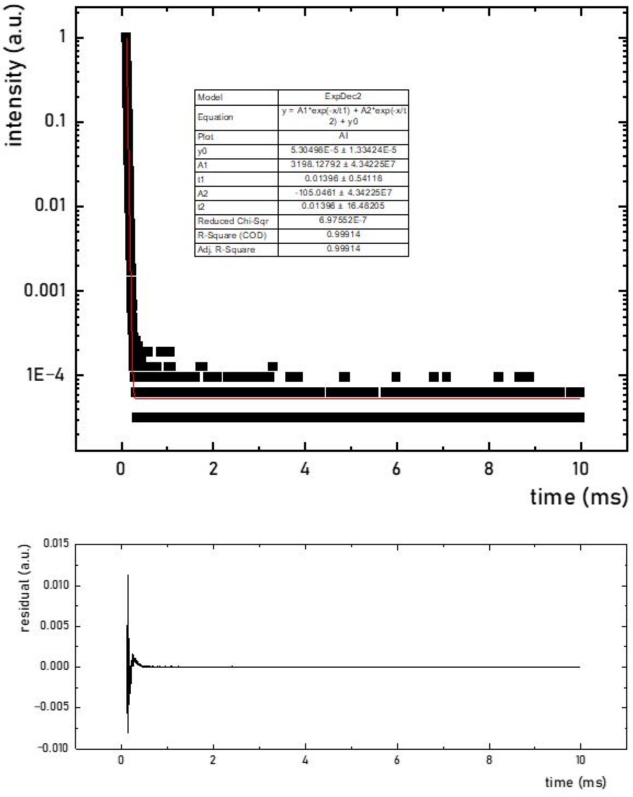

Figure S15. Luminescence decay with fitting curves for Ba_2LaNbO_6:1%Mn^{4+} (λ_{exc}=359 nm, λ_{em}=680.75 nm) measured at 403 K.

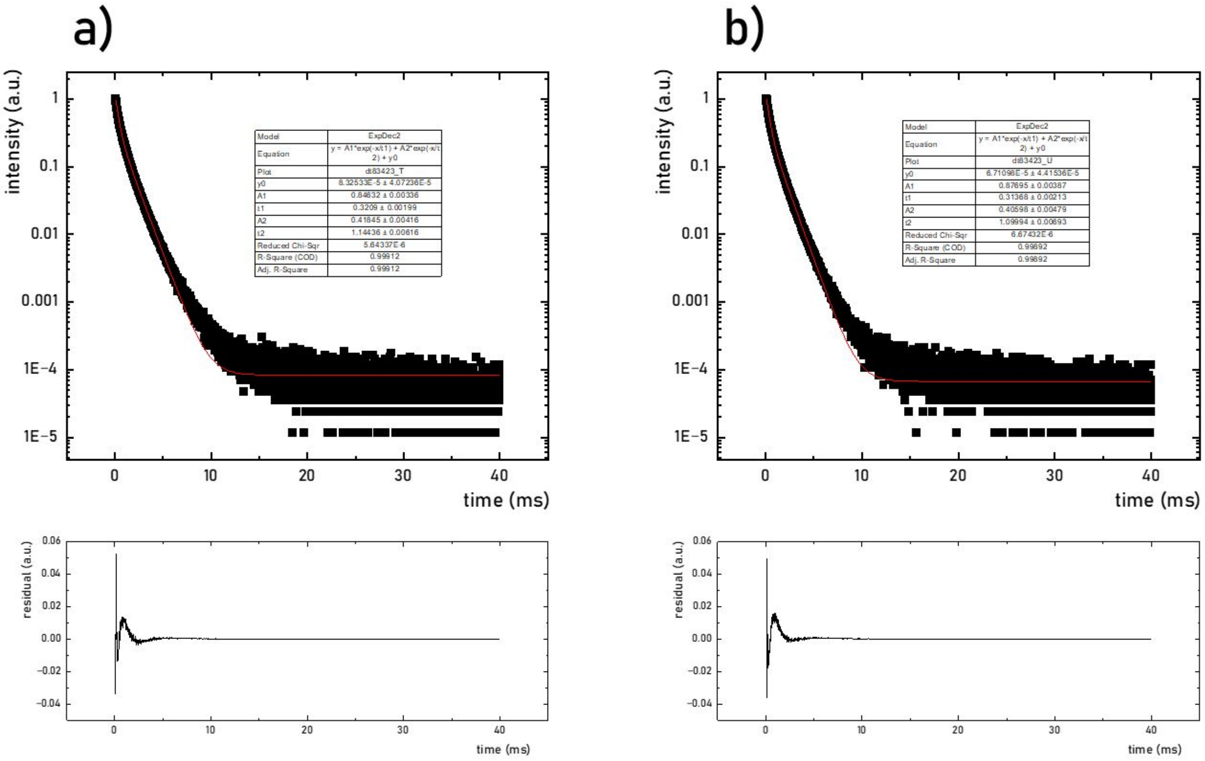

Figure S16. Luminescence decay with fitting curves for Ca_2LaNbO_6:1%Mn^{4+} (λ_{exc}=367.5 nm, λ_{em}=696.5 nm) measured at 83 K -a) and 103 K – b).

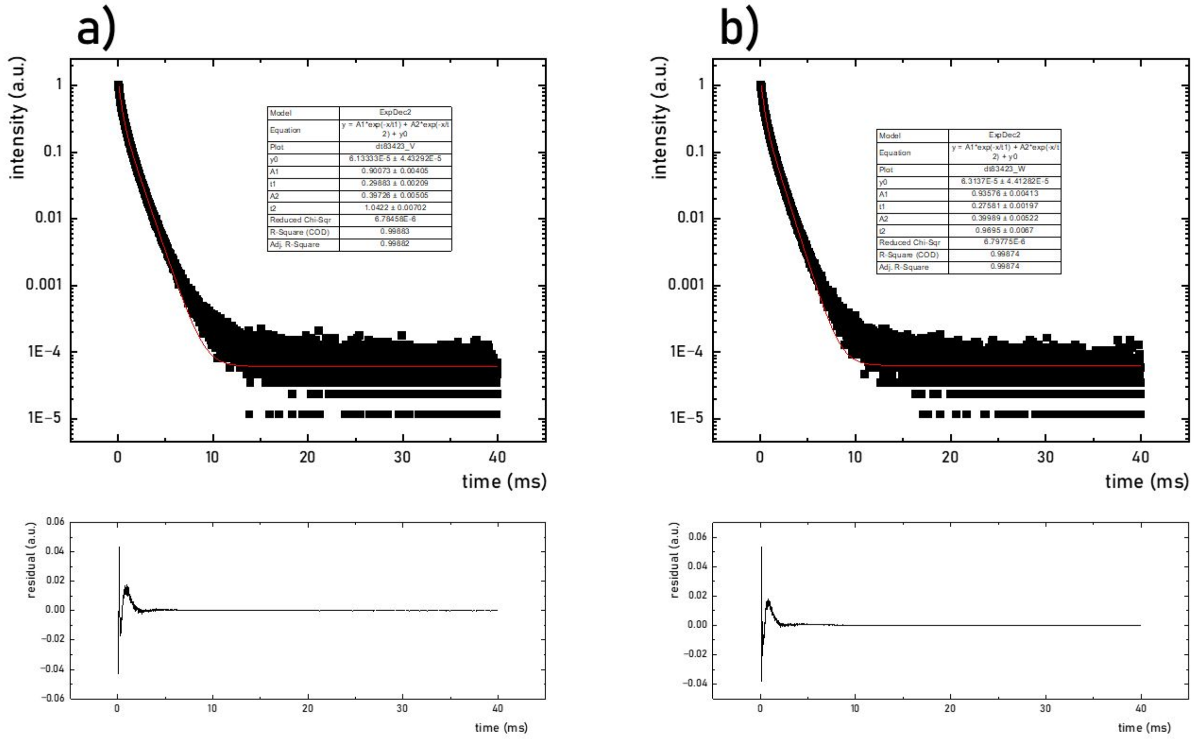

Figure S17. Luminescence decay with fitting curves for Ca_2LaNbO_6:1%Mn^{4+} (λ_{exc}=367.5 nm, λ_{em}=696.5 nm) measured at 123 K -a) and 143 K – b).

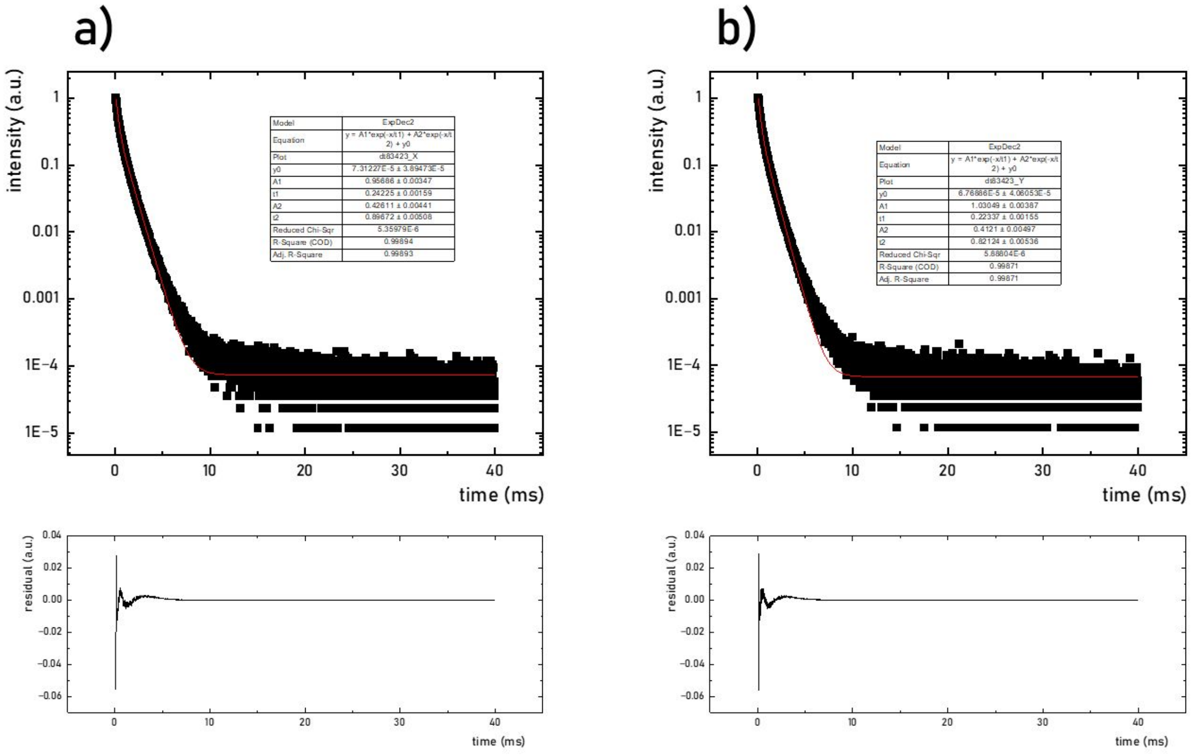

Figure S18. Luminescence decay with fitting curves for Ca_2LaNbO_6:1%Mn^{4+} (λ_{exc}=367.5 nm, λ_{em}=696.5 nm) measured at 163 K -a) and 183 K – b).

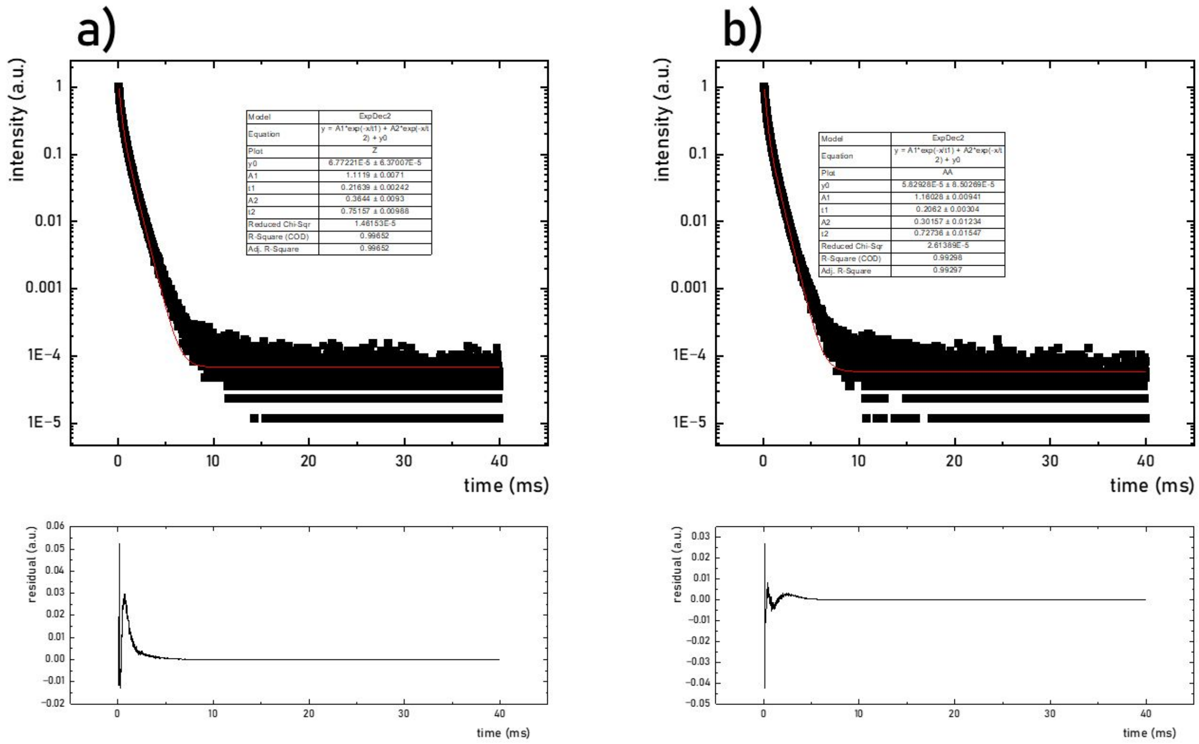

Figure S19. Luminescence decay with fitting curves for Ca_2LaNbO_6:1%Mn^{4+} (λ_{exc}=367.5 nm, λ_{em}=696.5 nm) measured at 203 K -a) and 223 K – b).

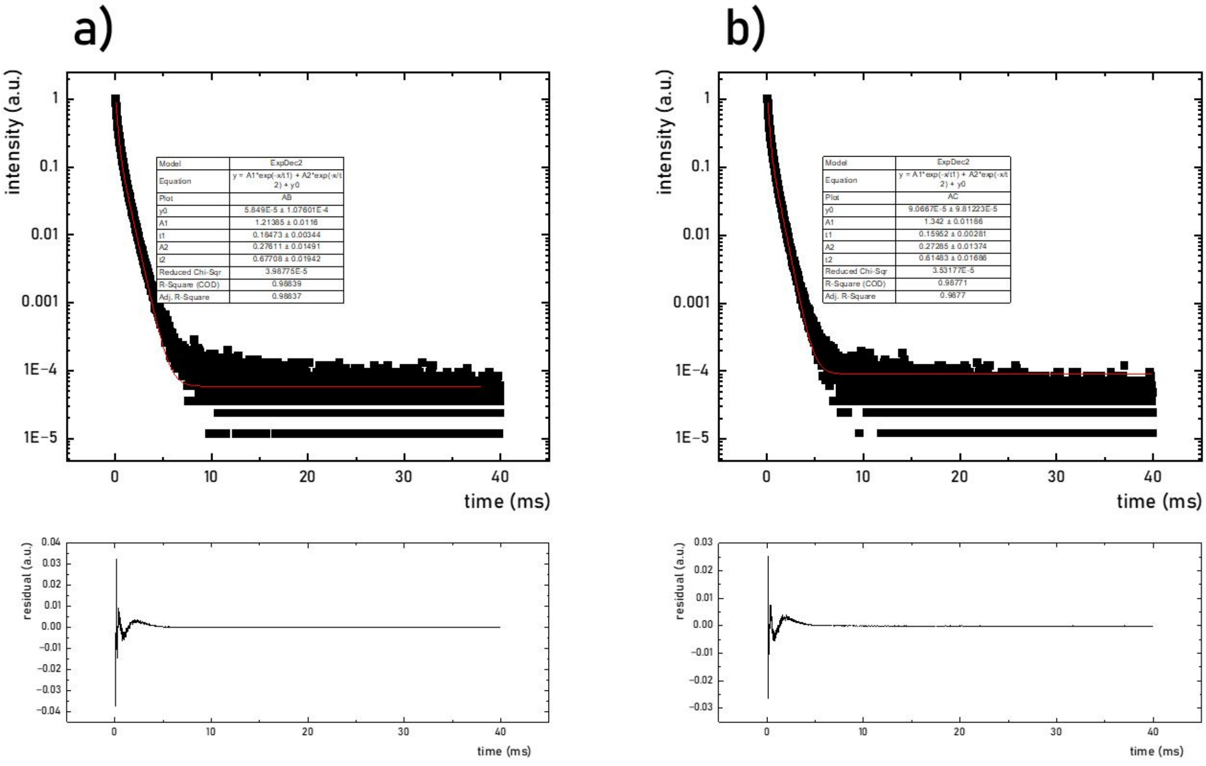

Figure S20. Luminescence decay with fitting curves for Ca_2LaNbO_6:1%Mn^{4+} (λ_{exc}=367.5 nm, λ_{em}=696.5 nm) measured at 243 K -a) and 263 K – b).

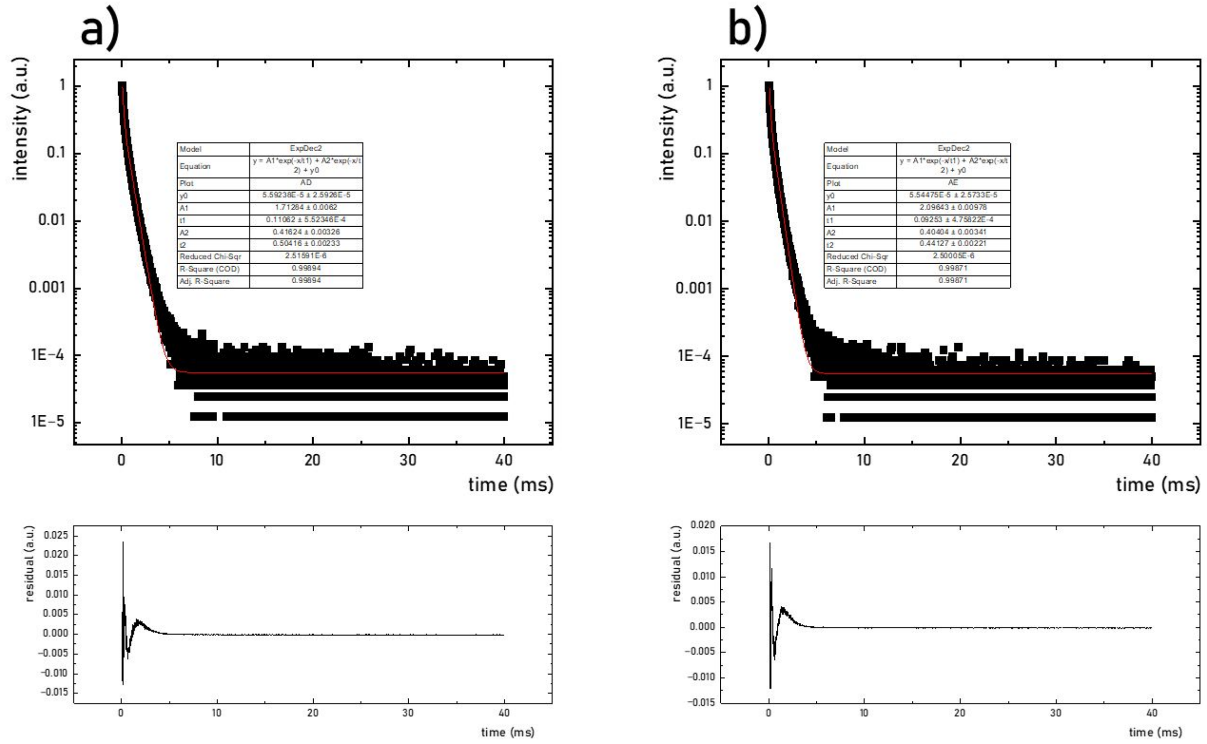

Figure S21. Luminescence decay with fitting curves for Ca_2LaNbO_6:1%Mn^{4+} (λ_{exc}=367.5 nm, λ_{em}=696.5 nm) measured at 283 K -a) and 303 K – b).

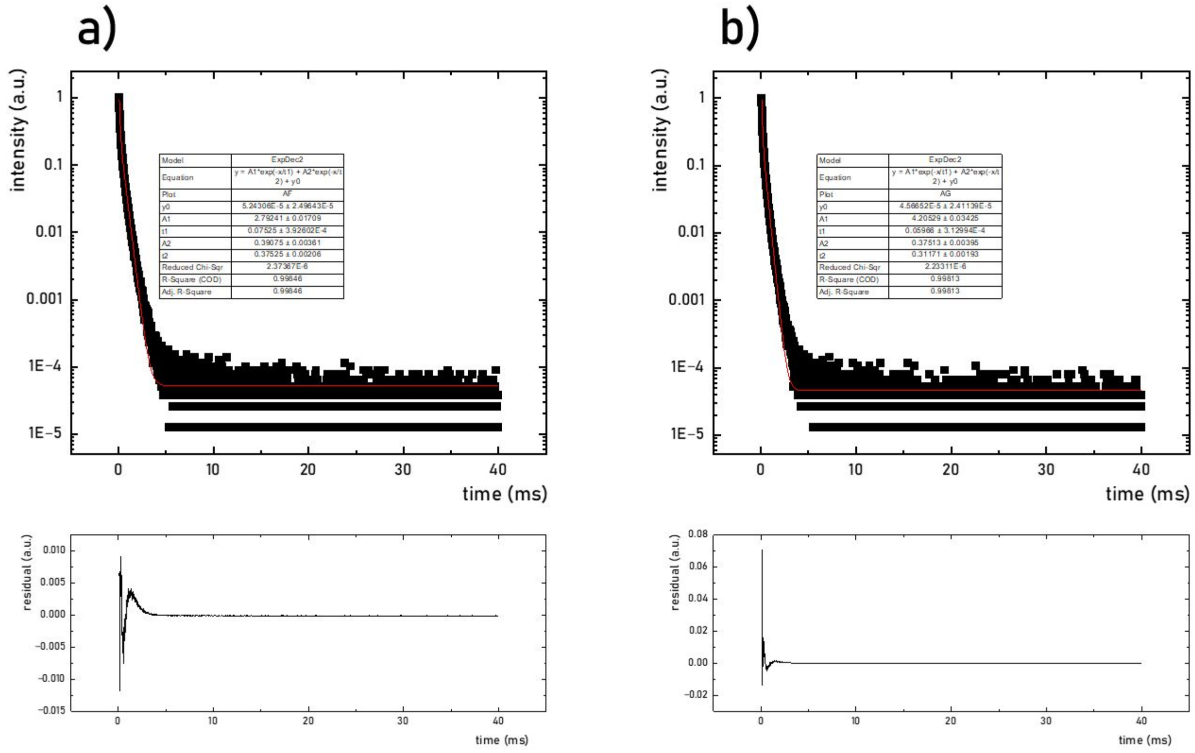

Figure S22. Luminescence decay with fitting curves for Ca_2LaNbO_6:1%Mn^{4+} (λ_{exc}=367.5 nm, λ_{em}=696.5 nm) measured at 323 K -a) and 343 K – b).

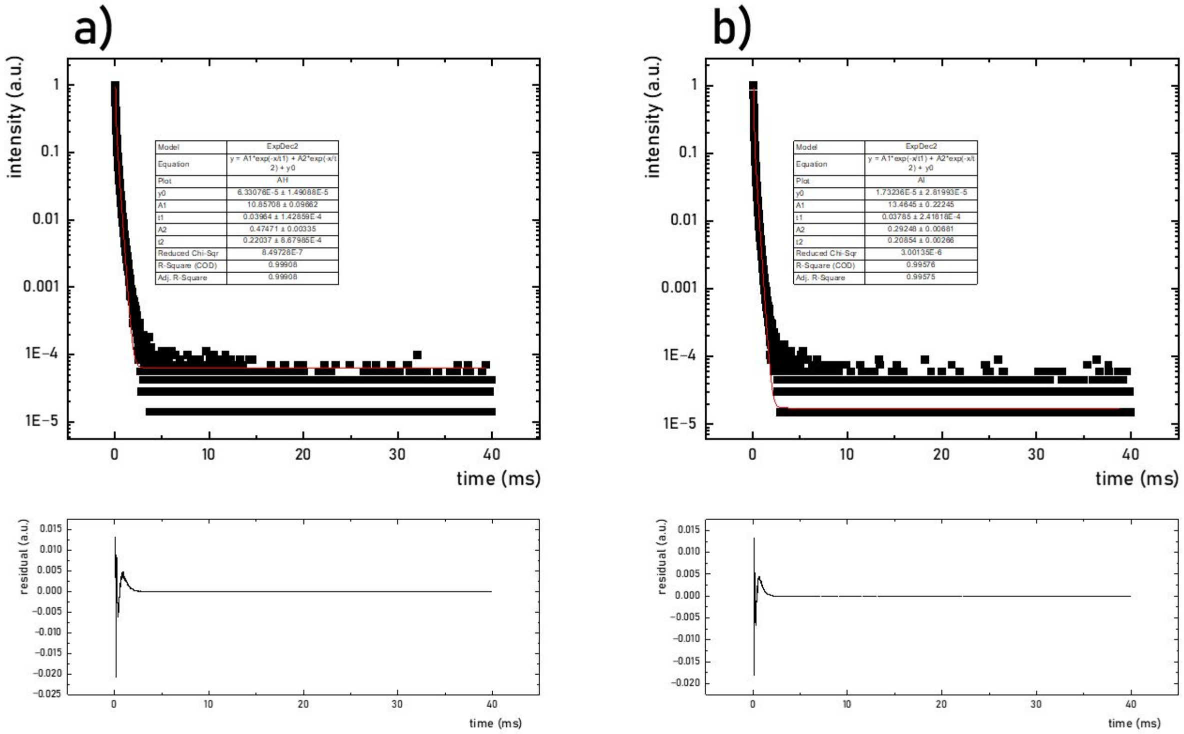

Figure S23. Luminescence decay with fitting curves for Ca_2LaNbO_6:1%Mn^{4+} (λ_{exc}=367.5 nm, λ_{em}=696.5 nm) measured at 363 K -a) and 383 K – b).

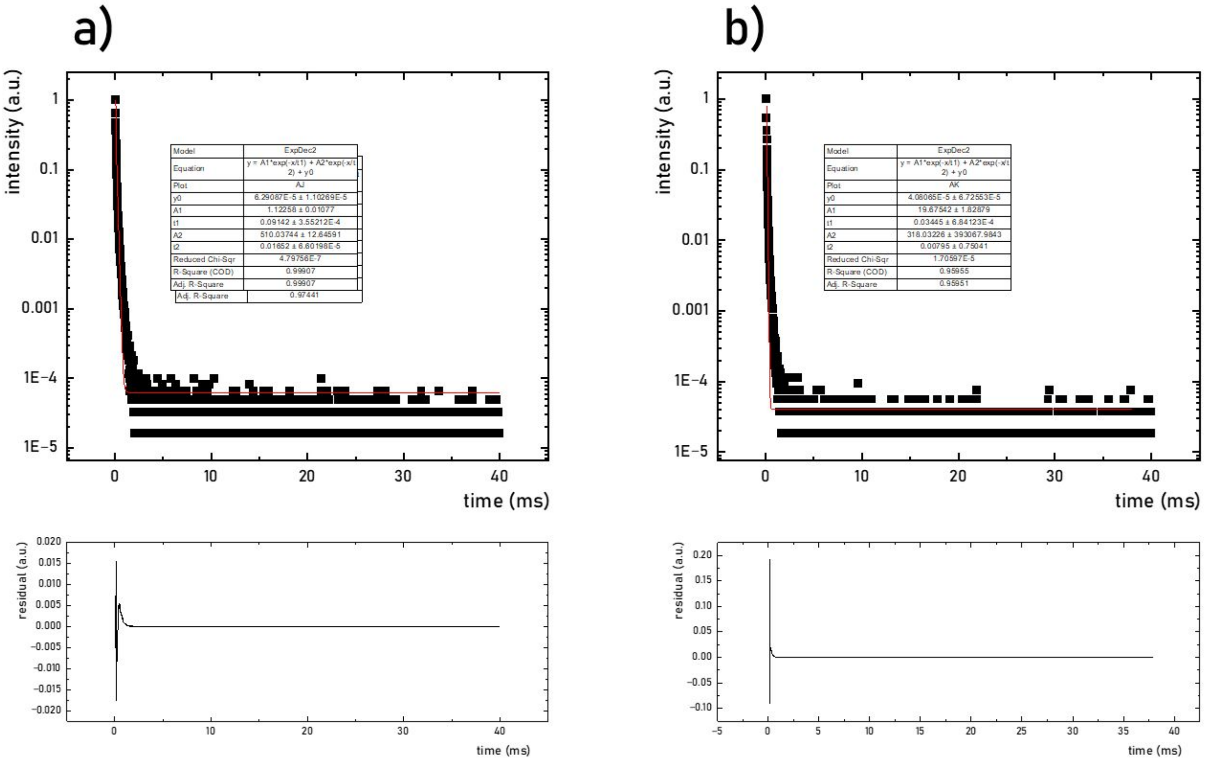

Figure S24. Luminescence decay with fitting curves for Ca_2LaNbO_6:1%Mn^{4+} (λ_{exc}=367.5 nm, λ_{em}=696.5 nm) measured at 403 K -a) and 423 K – b).

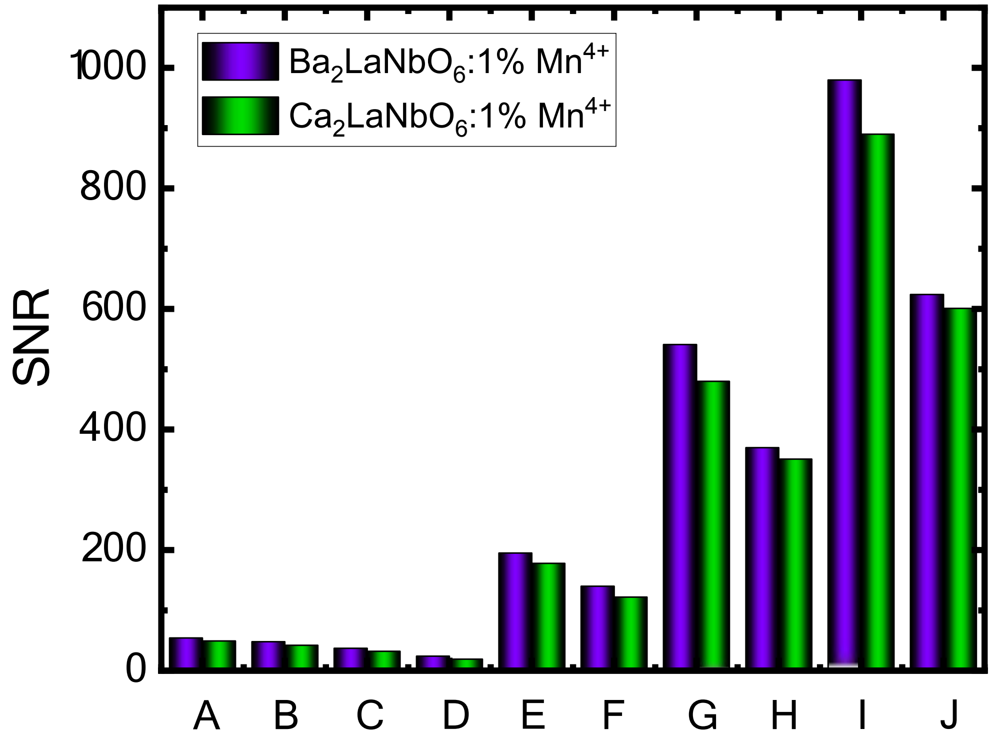

Figure S25. The Signal-To-Noise ratio (SNR) for the emission intensities of Ba_2LaNbO_6:1%Mn^{4+} of Ca_2LaNbO_6:1%Mn^{4+} integrated at different time gates calculated at 83 K.

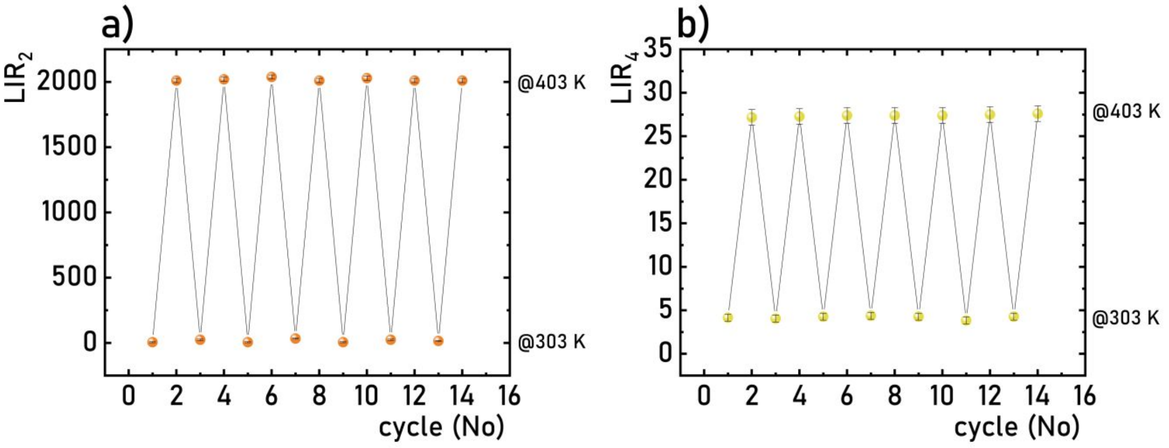

Figure S26. The LIR_2 for Ba_2LaNbO_6:1%Mn^{4+} -a) and LIR_4 for Ca_2LaNbO_6:1%Mn^{4+} -b) during heating and cooling cycles.

The δT can be determined using the following equation[1]:

$$\delta T = \frac{1}{S_R}\frac{\delta LIR}{LIR} \qquad \text{(S1)}$$

where $\delta LIR/LIR$ represents the uncertainty of LIR determination and was calculated as follows:

$$\frac{\delta LIR}{LIR} = \sqrt{\left(\frac{\delta I_1}{I_1}\right)^2 + \left(\frac{\delta I_2}{I_2}\right)^2} \qquad \text{(S1)}$$

where I_1 and I_2 represent the emission intensity used for LIR calculations. The δI is the uncertainty of the emission intensity - noise to signal ratio.

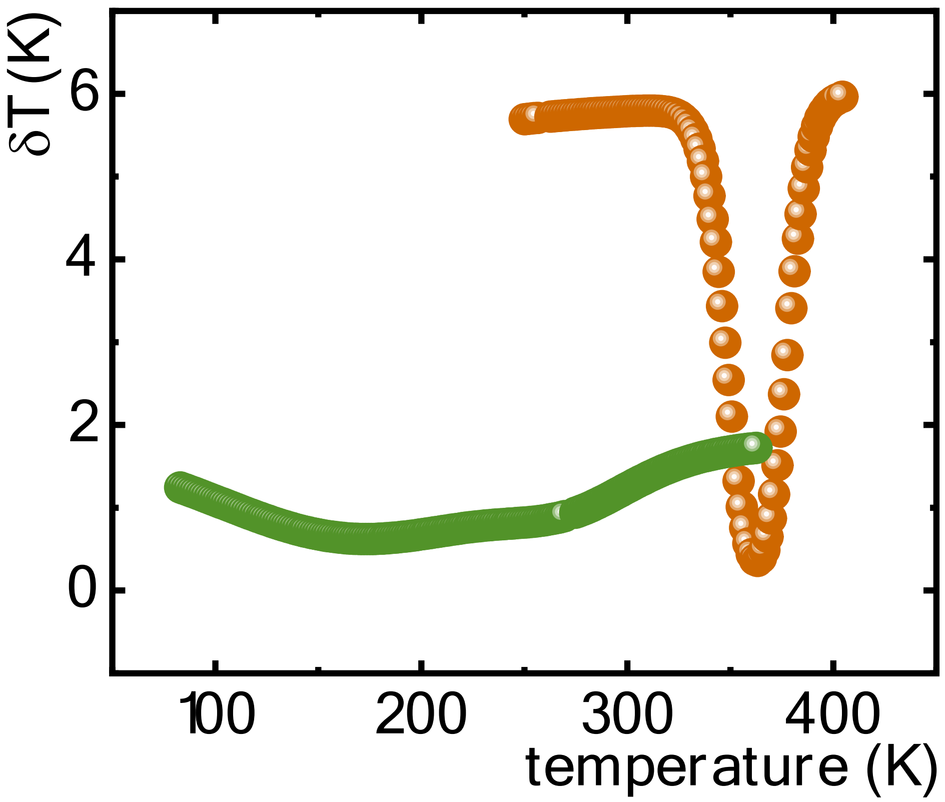

Figure S27. Thermal dependence of temperature determination uncertainties for LIR_2 (orange dots) and LIR_6 (green dots) for Ba_2LaNbO_6:1%Mn^{4+}.

References:

[1] M. Back, J. Ueda, M. G. Brik, T. Lesniewski, M. Grinberg and S. Tanabe, *ACS Appl Mater Interfaces*, 2018, **10**, 41512–41524.
